# Proteogenomic profiling of soft tissue leiomyosarcoma reveals distinct molecular subtypes with divergent outcomes and therapeutic vulnerabilities

**DOI:** 10.1101/2025.11.19.689365

**Published:** 2025-11-23

**Authors:** Atsushi Tanaka, Makiko Ogawa, Yusuke Otani, Ronald C. Hendrickson, Zhuoning Li, Narasimhan P. Agaram, David S. Klimstra, Julia Y. Wang, Michael H. A. Roehrl

**Affiliations:** 1 Department of Pathology, Beth Israel Deaconess Medical Center, Harvard Medical School, Boston, MA, USA; 2 Broad Institute of MIT and Harvard, Cambridge, MA, USA; 3 Former address: Department of Pathology and Laboratory Medicine, Memorial Sloan Kettering Cancer Center, New York, NY, USA; 4 Department of Biochemistry and Molecular Biology, Miller School of Medicine, University of Miami, Miami, FL, USA; 5 Sylvester Comprehensive Cancer Center, Miller School of Medicine, University of Miami, Miami, FL, USA; 6 Former address: Sloan Kettering Institute, Memorial Sloan Kettering Cancer Center, New York, NY, USA; 7 Sloan Kettering Institute, Memorial Sloan Kettering Cancer Center, New York, NY, USA; 8 Department of Pathology and Laboratory Medicine, Memorial Sloan Kettering Cancer Center, New York, NY, USA; 9 Department of Pathology, Yale University School of Medicine, New Haven, CT, USA; 10 Curandis, Boston, MA, USA; 11 Wyss Institute at Harvard, Boston, MA, USA; 12 Lead contact

## Abstract

Soft tissue leiomyosarcoma (STLMS) is an aggressive malignancy lacking validated molecular subclassification and effective targeted treatments. We performed comprehensive proteogenomic analysis of primary and metastatic STLMS to uncover biological traits and therapeutic weaknesses. Integrative proteomic and phosphoproteomic analyses using non-negative matrix factorization identified three subtypes. Subtype P1 shows genomic stability, low proliferation, and enrichment of FGFR2 and PDK signaling pathways. Subtype P2 exhibits chromosomal instability, inflammatory programs, activation of CDK-AURKA/B-mTOR/ERK kinome with *IGF1R*/*PDGFRA* gene alterations, and poorest survival outcomes. Subtype P3 is highly proliferative, with E2F/DNA-repair programs, elevated NCOR1, and shift towards nonhomologous end joining with upregulation of PARP1. Homologous recombination deficiency (HRD) analysis distinguishes HRD-low P1 from HRD-high P2/P3. Paired analyses suggest HRD increases in metastases within P3. Immune profiling shows P2 as immunosuppressive, characterized by LGALS9 and M2 macrophages. Our proteogenomic analyses provide a molecular landscape of LMS, revealing biological insights, patient outcome stratification, and therapeutic targets.

## Introduction

Soft tissue sarcomas (STS) are a diverse group of mesenchymal malignancies that include over 80 histological subtypes^1^. Leiomyosarcoma (LMS) accounts for 10-20% of all STS^2^. Soft tissue leiomyosarcoma (STLMS), i.e., extra-uterine LMS, develops mainly in the retroperitoneum, abdomen/pelvis, trunk, and extremities, with the first two being more common^3^. Clinical management of localized disease depends on anatomical site and tumor grade^4,5^. Typical STLMS shows spindle-shaped cells that are set in long intersecting fascicles, diffuse hypercellularity with high-grade histology, and the expression of one or more myogenic marker proteins. Despite multidisciplinary management, cure rates for STLMS remain low. Half of patients experience tumor relapse after surgery^6-8^. Patients with advanced or metastatic STLMS have poor outcomes and limited treatment options^9,10^. To improve outcomes, there are urgent needs to develop better classification and prognostic stratification of STLMS patients for the development of efficient therapeutics.

Several genomic and epigenomic studies of pan-STS reported enrichment of PIK3/AKT pathway alterations and deletions of tumor suppressor genes, including *TP53* (9% deep and 60% shallow deletions), *RB1* (14% deep, 78% shallow), and *PTEN* (13% deep, 68% shallow)^11-13^. Multiomic analyses showed that LMS is molecularly distinct from other STS, and that STLMS differs from uterine LMS in hormone response and DNA damage response pathways. Integrated clustering of STLMS based on WES, epigenome, and whole transcriptome data revealed two subtypes: one with short survival and frequent PI3K/AKT/MTOR pathway alterations, the other with longer survival and minimal PI3K/AKT/MTOR alterations. While MTOR inhibitors like everolimus or temsirolimus show some clinical efficacy in LMS^14,15^, their efficacy is reduced by indirect AKT upregulation.

Proteins are the “machines of life” carrying out complex cellular and biochemical functions and thus serve as cancer treatment targets^16^. mRNA abundance poorly predicts protein amounts^17,18^. Proteomics complements genomic research by providing accurate assessment of tumor biology and patient stratification that is inaccessible to nucleic acid-based approaches. Two proteomic analyses of pan-STS were recently published, although the use of formalin-fixed, paraffin-embedded samples limits proteomic studies and largely precludes analyses of posttranslational modifications, such as phosphorylation^19,20^. Here, we present the first integrative proteogenomic study of STLMS using freshly frozen tissue and the inclusion of phosphoproteomics to discover alterations of the kinome.

To gain a better understanding of STLMS tumor biology with focus on associations between genomic alterations and both total proteome and phosphoproteome, we analyzed 72 primary site and metastatic STLMS samples, including 18 paired primary-metastasis lesions. Additionally, we incorporated our own independent validation cohort of 222 STLMS FFPE samples as tissue microarrays.

## Results

### Unsupervised clustering of proteome identifies 3 proteomic subtypes with clinical relevance

**Figure 1.**
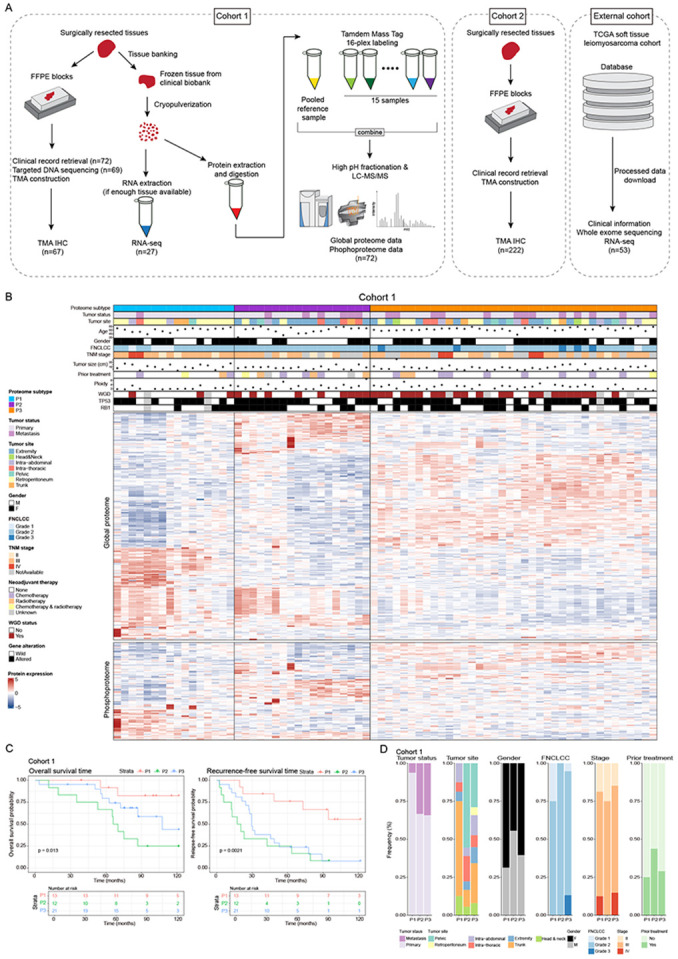
Integrative study design and clinicopathological features of proteome subtypes (A) Summary workflow and metrics of this study (B) Summary plot of cohort 1 including clinicopathological information and proteome subtypes. In the heatmap, proteins and phosphoproteins which show significant differences (FDR <0.05) between proteome subtypes are shown. (C) Kaplan-Meier curves of overall survival and recurrence-free survival between proteome subtypes (D) Differences of clinicopathological factors between proteome subtypes

We obtained 72 frozen tissues of primary and metastatic STLMS (cohort 1) for liquid chromatography-mass spectrometry (LC-MS) analysis (Figure 1A). For independent cohort 2, we obtained FFPE blocks of 222 STLMS cases and constructed tissue microarray blocks (Figure 1A). Patient demographics are summarized in Table S1. LC-MS analysis of global proteomes quantified 10,249 proteins in total and 7,325 proteins in at least 30% of samples (Table S2A). For phosphoproteomics, the analysis quantified 6,542 phosphorylated proteins in total and 4,584 in at least 30% of samples (Figure S1A, Table S2B). Batch correction removed TMT multiplexing effects from the data (Figure S1B). Using non-negative matrix factorization (NMF) on filtered global proteome and phosphoproteome data, we identified three distinct proteome-based subtypes (P1, P2, and P3) of STLMS (Figures 1BC, S1CD). Survival analyses showed significant differences in overall survival time (OS) and recurrence-free survival time (RFS) between subtypes, with P2 showing the shortest patient survival. We validated these findings using a TCGA STLMS dataset, where P2/P3 subtypes showed significantly shorter recurrence-free survival and overlapped with the TCGA STLMS iCluster1 subtype (Fig. S1EF). Analysis of clinical attributes (Figure 1D) revealed no significant differences in tumor size, age, tumor status (primary lesion or metastatic lesion), gender, TNM stage, or prior treatment between proteomic subtypes. However, metastatic samples were enriched in P2/P3 subtypes compared to P1 (p=0.0308). Primary tumor site distribution varied significantly among subtypes (p=0.01735), with trunk samples predominantly in P1 and pelvis samples in P2. FNCLCC Grade 1 tumors were only seen in P1, while FNCLCC Grade 3 tumors were only seen in P3.

### Differences of proteogenomic landscape and transcription regulatory networks between proteome-based subtypes

**Figure 2.**
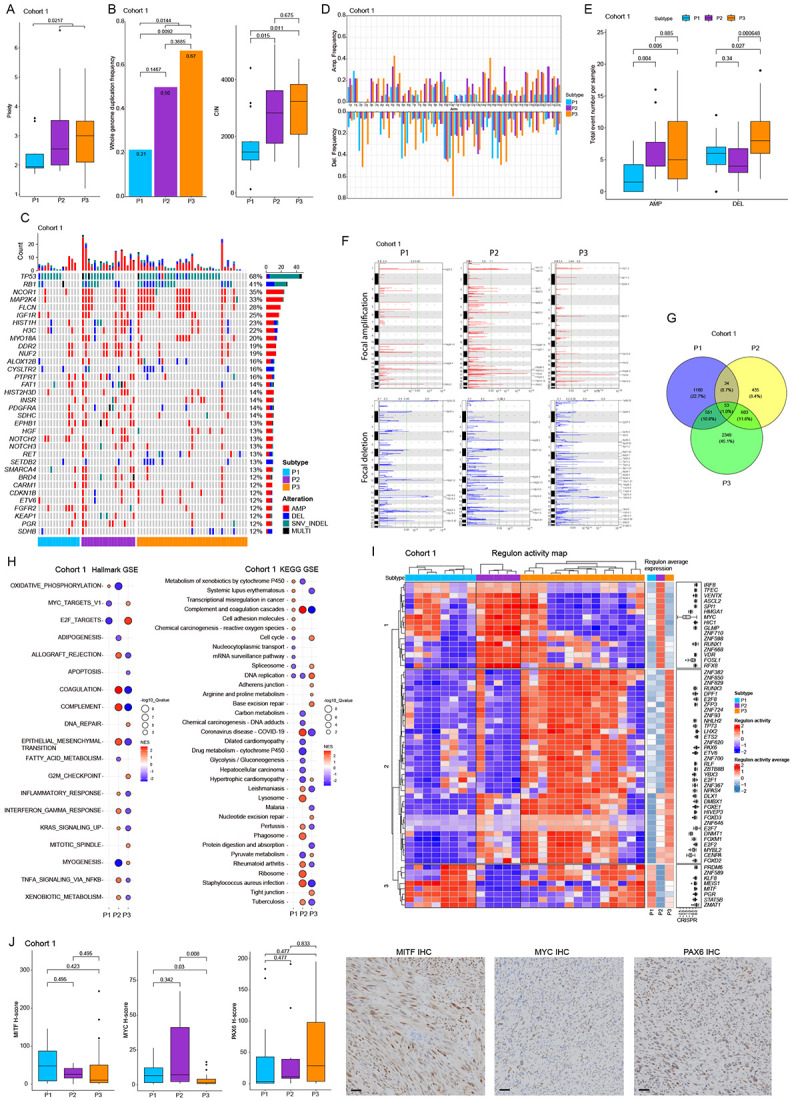
Landscape of genomic alterations and regulatory activity across proteome subtypes (A) A boxplot showing ploidy between subtypes. P2/P3 show significantly higher ploidy than P1. (B) Bar chart of whole genome duplication (WGD) frequency in each subtype on the left and boxplot of chromosomal instability on the right. P2/P3 show significantly higher frequency of WGD and a higher CIN score than P1. (C) Oncoprint of integrated genomic alterations including SNV, Indel, and CNA. Genes with 8 events across cohort 1 (>12% samples) are shown. The bar chart on the right side of the oncoprint shows the alteration frequency across cohort 1. The bar chart on the top shows the number of genomic events of each sample. (D) Bar plot showing arm-level amplifications and deletions. In general, P1 shows lower frequency of arm-level events for each chromosome. P3 shows the highest frequency of arm-level deletions for most chromosomes. (E) Concordant with arm-level event frequencies for each chromosome, total event numbers of amplifications are significantly higher in P2/P3 than in P1. For arm-level deletions, P3 shows the highest event numbers compared with P1 or P2. (F) Focal amplification/deletion patterns in each subtype are shown. P1 has a relatively lower number of focal peak events. The 17p12 peak (NCOR1 and MAP2K4 gene loci) are shared between the P2 and P3 subtypes. (G) Venn diagram showing overlap of genes in in the recurrent focal peaks of each subtype. (H) Balloon plot showing pathway enrichment analyses of Hallmark and KEGG terms between subtypes. Only statistically significant results are shown. (I) Regulatory activity profile of STLMS by subtype status based on transcriptome data. Computed regulon activity scores are shown as a heatmap. Regulons with p<1E-8 from the Master Regulator Analysis process are shown. The boxplot on the right side of the heatmap shows DepMap CRISPER gene effect scores for 36 sarcoma cell lines. (J) Boxplots of MYC, PAX6, MITF IHC scores by subtypes are shown. In addition, representative IHC images are shown. Each transcription factor shows an expression profile of subtypes that is concordant with regulon network analyses of transcriptome data. Bars, 50 μm.

We evaluated genomic alterations in 69 samples by MSK-IMPACT targeted sequencing. P2/3 subtypes showed higher ploidy, whole genome duplication, and chromosomal instability versus P1 (Figure 2AB, findings supported by analysis of the TCGA STLMS cohort (Figure S2AB). Genomically, *TP53* (68%) and *RB1* (41%) were most frequently altered, followed by *NCOR1* (35%), *MAP2K4* (33%), and *FLCN* (28%) (Figure 2C). The GENIE STLMS cohort (v15.0, n=774)^21^ showed similar frequencies. P2/P3 showed frequent genomic alterations in *NCOR1*, *MAP2K4*, *FLCN*, and *IGF1R*. P2 showed commonly alterations in oncogenic drivers like *IGF1R* or *PDGFRA* and SWI/SNIF complex genes, associated with poor outcomes^22-25^. Somatic copy number alteration (SCNA) analysis revealed more arm-level amplifications in P2/P3 than in P1, while P3 showed higher deletion frequencies (Figure 2DE). Each subtype showed unique focal amplification/deletion patterns, with P1 showing fewer focal peaks (Figure 2F). The 17p12 peak, containing *NCOR1* and *MAP2K4*, was shared between P2 and P3. *NCOR1* and *MAP2K4* alterations occurred frequently together (p=1.03E-14, Fisher’s exact test). We analyzed gene overlap in recurrent focal peaks, finding 1,818, 1,125, and 3,556 involved genes respectively (Figure 2G). Gene ontology analyses showed P1 focal deletion peaks enriched in copper/zinc ion processes, P2 focal peaks in extracellular matrix processes, and P3 focal peaks in T-cell receptor signaling (Figure S2C). KEGG analysis showed P2/P3 deletion peaks enriched in immune-related pathways, while only P3 showed deletions involving in PI3K-Akt signaling (Figure S2D).

Pairwise differential expression analyses between subtypes (P1 vs. P2/P3, P2 vs. P1/P3, P3 vs. P1/P2) revealed 2,277 dysregulated proteins in P1 vs. others (207 upregulated >2-fold, 211 downregulated >2-fold), 2,503 in P2 vs. others (282 upregulated >2-fold, 286 downregulated >2-fold), and 2,652 in P3 vs. others (106 upregulated >2-fold, 377 downregulated >2-fold) with q-values <0.05 (Figure S2E) (Table S3ABC). KEGG and Hallmark analyses identified cancer-related pathways in each subtype (Figure 2H). P1 showed low proliferation and enriched metabolic processes. P2 showed enrichment of epithelial-mesenchymal transition (EMT), NF-kB pathway, inflammatory responses, and KRAS signaling, with downregulated oxidative phosphorylation. P3 exhibited cell cycle activation, DNA repair, low EMT, and higher muscle differentiation. Transcriptome regulatory network analysis identified 60 dysregulated regulons across proteome subtypes (Figure 2I). The regulons formed 3 clusters. Cluster 1 showed high activity in the P2 subtype with poor outcome (Figure 1B), with MYC enrichment confirmed by IHC (Figure 2J). Cluster 2 showed high activity in P3, with enriched cell cycle-related transcription factors. DNMT1 enrichment in P3 suggests methylation differences. PAX6, a tumor progression factor^26-28^, was upregulated in P3 and confirmed by IHC (Figure 2J). Cluster 3 showed high activity in P1 and some P3 samples. MITF, regulating immune response^29^, was enriched in P1 (Figure 2J) and validated in the TCGA STLMS dataset (Figures 2J, S2F).

### Impact of genomic alterations on proteomic subtypes and cell cycle-related components

**Figure 3.**
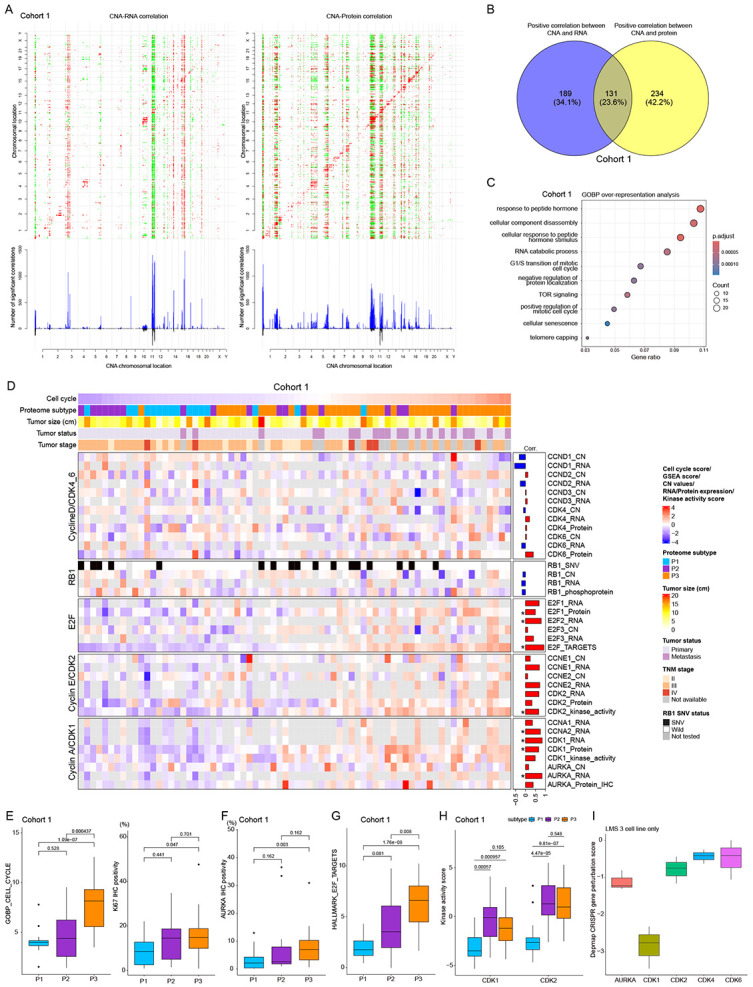
Impact of genomic alterations on the proteome (A) The correlations between CNA and mRNA (left) and protein expression (right) levels (y axes) reveal both cis and trans effects. The presence of significant positive (red) and negative (green) Spearman’s correlations (FDR <0.1) between CNA and mRNA or protein is highlighted. The numbers of significant cis and trans events corresponding to the indicated genomic loci specific to mRNAs or proteins are represented by blue bars, while the overlap between CNA-mRNA and CNA-protein events is depicted by black bars. (B) Venn diagram showing cis correlation overlap between CNA-mRNA and CNA-protein. (C) GOBP over-representatation analysis of cis correlation between CNA-protein is shown. Cell cycle-related terms are significantly enriched. (D) Genomic effect of cell cycle-related genes on mRNA and protein. Columns represent each sample and are ordered by cell cycle score. The bar chart on the right side of heatmap shows Spearman’s correlation coefficients between targets and cell cycle score. “E2F targets” is based on ssGSEA of the Hallmark E2F term. Kinase activity scores (CDK1,2 kinase activities) are from PTM-GSE analysis. p_RB1 denotes phosphorylated protein of RB1. * denotes statistical significance (p<0.05). (E) Boxplots showing proteome subtype differences of GOBP cell cycle scores and Ki67-positive tumor frequency. (F) Boxplot showing AURKA protein expression (IHC score) by subtypes. (G) Boxplot of E2F scores from ssGSEA by subtypes. (H) Boxplot showing kinase activity scores of CDK1/2 by subtypes. (I) Boxplot of Depmap CRISPR gene perturbation scores targeting CDKs and AURKA using 3 leiomyosarcoma cell lines. Score below −1 represents significant effect on cell survival when the target gene is inhibited.

We studied cis effects of copy number alteration (CNA) on mRNA and protein. Analysis found 320 and 365 cis correlations of CNA-mRNA and CNA-protein respectively (FDR <0.1) (Figure 3A), with 131 genes overlapping (Figure 3B). GOBP over-representation analysis of cis regulated genes that showed positive CNA-protein abundance correlations revealed significant enrichment of cell cycle-related terms (Figure 3C). Most cell cycle components showed positive correlation with the cell cycle score at CNA, mRNA, and (phosho)protein levels (Figure 3D). The P3 subtype showed highest cell cycle activity, Ki67 immunolabeling (Figure 3E), and AURKA protein expression (Figure 3F), an emerging cancer treatment target^30^. E2F targets were enriched in P2/P3 (Figure 3G), with CDK1/2 kinase activity significantly higher than in P1 (Figure 3H). Independent TCGA STLMS data validated these findings (Figure S3A-C). CRISPR-Cas9 gene perturbation experiments of the DepMap project showed CDK1 and AURKA inhibition greatly affected cell survival (Figure 3I), suggesting that these proteins are potential treatment targets for high proliferation P3 subtype STLMS. Metastatic STLMS samples showed increased cell cycle score, proliferation rate, and CDK2 kinase activity relative to primary site STLMS (Figure S3DE), with a trend for elevated AURKA protein expression (Figure S3F). TMA cohort 2 IHC scores validated these findings (Figure S3G). Combined cohort analyses of recurrence-free survival showed poor outcomes associated with increased expression of cell cycle-related components (Figure S3H), supporting AURKA and CDKs as potential therapeutic targets against STLMS including metastatic lesions.

### Phosphoproteomic alterations associated with proteomic subtypes and receptor tyrosine kinase pathways

**Figure 4.**
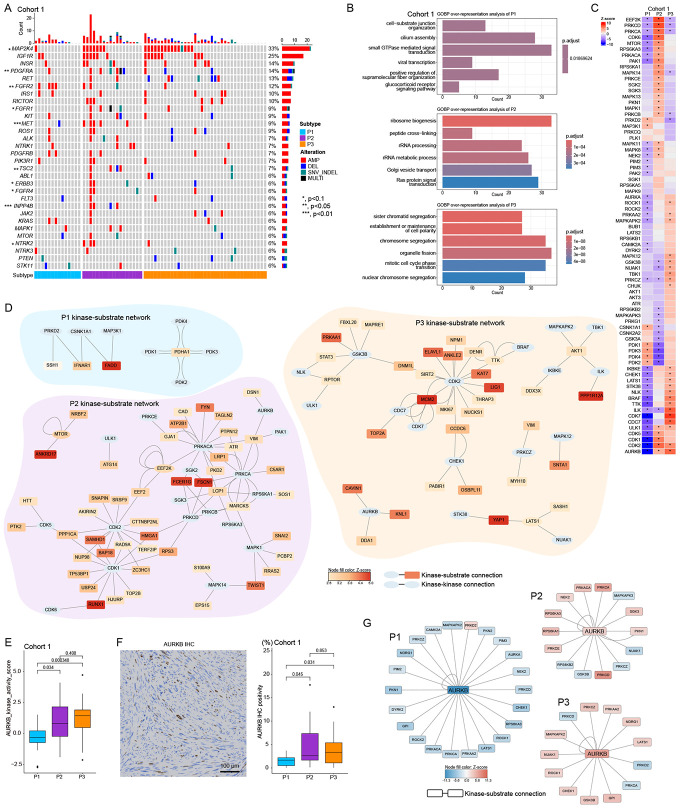
Phosphoproteomic differences between subtypes (A) Oncoprint showing RTK-RAS pathways with 4 or more events. Amplification of MAP2K4 and IFG1R are the top 2 most frequent alterations. * denotes statistically significant differences (Fisher’s exact test) in gene alteration frequency between subtypes. (B) Over-representation analyses of GOBP terms. Phosphoproteins which are positively upregulated in one subtype compared to other subtypes with FDR<0.05 are used for GOBP term enrichment analyses. (C) Kinase activity heatmap by subtypes. Z-scores of inferred kinase activities are plotted as a heatmap. * denotes statistical significance. Kinase names are shown on the left side of the heatmap. (D) Kinase-substrate networks of the top 20 positively enriched kinases in each subtype are shown. Note: all positively enriched kinases of P1 are shown due to its small number. Genes shown as ellipses are kinases. Genes shown as rectangles are kinase substrates Colors of kinase substrates denote the phosphorylation Z-score of the target’s phosphorylation site. If multiple phosphorylation sites exist in a substrate, phosphorylation averages are shown based on color scale. (E) Boxplot of PTM-GSEA AURKB activity score shows enrichment in P2/P3 compared to P1. (F) Representative AURKB IHC image and boxplot of AURKB IHC score by subtype. Bar, 100 μm. (G) Networks of AURKB and inferred regulatory kinases are shown. Motif analysis of AURKB-T232 identifies 194 kinases. Among them, kinases with significant enrichment (FDR<0.05) in RoKAI app analysis between subtypes were selected to be shown here. Colors of kinases denote Z-scores of kinase activity.

We investigated phosphoproteomic differences between STLMS subtypes focusing on receptor tyrosine kinase molecules (RTK-RAS pathways). Among 69 DNA-sequenced samples, 56 samples (81.2%) had at least one genomic alteration in RTK-RAS pathway genes. *MAP2K4* and *IGF1R* were the most frequent events. Though alteration frequencies were higher in P2/P3 vs. P1, differences were not statistically significant (Figure 4A). Genomic alteration frequencies of *PDGFRA* (overall frequency,14%), *FGFR2* (12%), *MET* (9%), *TSC2* (7%), *ERBB3* (6%), *FGFR4* (6%), *INPP4B* (6%) differed significantly between proteomic subtypes. All these genes except *FGFR2* showed enrichment in P2, while *FGFR2* was enriched in P1. Analyses of differential expression at phosphoproteome level between subtypes revealed Ras protein signal transduction enrichment in P2 and cell cycle term enrichment in P3 (Figure 4B), compatible with RTK-RAS gene enrichment in P2 and cell cycle enrichment in P3 (as shown in Figures 2 and 3). We profiled kinase activity using phosphopeptide expression data and the RoKAI app^31^ to generate kinase-substrate networks (Figure 4CD). CSNK1A1 and PDK1-4 were enriched in P1. AURKA activity was downregulated in P1, matching AURKA IHC assessment (Figure 3F). CDKs (CDK1,2,5,6), AURKB, mTOR, and MAPK1 (ERK2) were enriched in P2. CDKs (CDK2,7), AURKB, ROCK1, and BRAF were enriched in P3. AKT1/3 showed enrichment in P3 versus P1/P2, though not statistically significant. P1 had a distinct kinase activity profile from P2/P3, while P2/3 shared kinase activities such as AURKB or CDK2. This difference may derive from distinct genomic alteration patterns (Figure 4A). PTM-GSEA analyses showed similar kinase activity differences between STLMS subtypes (Figure S4A). AURKB kinase activity scores were higher in P2/P3 versus P1 (Figure 4E). Furthermore, AURKB IHC positivity was higher in P2/P3 compared to P1 (Figure 4F). Consistent with this observation, AURKB mRNA expression in the independent TCGA cohort was also higher in P2/P3 versus P1 (Figure S4B). The most frequent AURKB phosphorylation site with activating effect is T232 based on the PhosphositePlus database (https://www.phosphosite.org/homeAction). Motif analysis of AURKB-T232 predicted responsible kinases for phosphorylation and inferred AURKB upstream regulators (Figure 4G). Most inferred upstream kinases showed activation in P2/P3, while AURKB-activating kinases were downregulated in P1.

### NCOR1 protein expression is associated with smooth muscle differentiation, cancer stemness, and immune-suppressive phenotype

**Figure 5.**
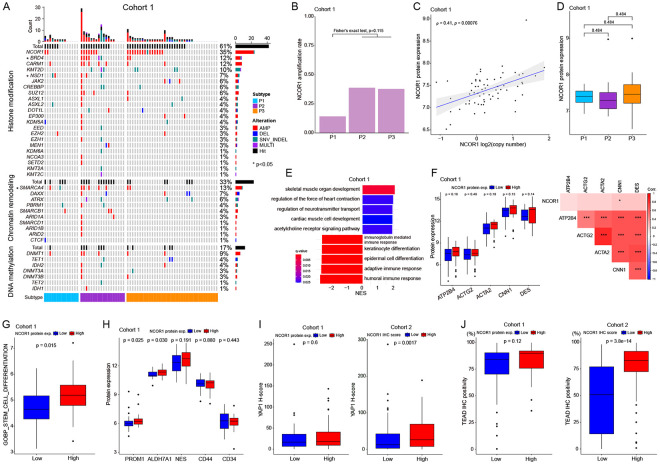
NCOR1 expression corelates with muscular differentiation, stemness, and immune status (A) Oncoprint of epigenetic regulators in cohort 1. NCOR1 gene is the top altered epigenetic regulator. * denotes statistical difference of gene alteration frequency between subtypes. (B) NCOR1 amplification shows higher frequency in P2/P3 compared to P1 in cohort 1 (butnot statistically significant). (C) NCOR1 copy number positively correlates with its protein level (Spearman's correlation coefficient) in cohort 1. (D) Boxplot of NCOR1 protein expression by subtypes in cohort 1. (E) Top 5 positively and negatively enriched GOBP GSEA results are shown. A ranked gene list based on fold change values between NCOR1-low and -high groups in cohort 1 was used for GSEA. (F) Boxplot of smooth muscle protein markers between NCOR1-low and NCOR1-high. (G) Boxplot of GOBP_STEM_CELL_DIFFERENTIATION ssGSEA scores. In cohort 1, the NCOR1-high group shows significantly higher scores compared to the NCOR1-low group. (H) Boxplot of stemness markers between NCOR1-low/high groups. PROM1 (CD133) and ALDH7A1 in the NCOR1-high group show significantly higher protein expression than the NCOR1-low group in cohort 1. (I) Boxplots of YAP1 IHC H-scores between NCOR1-low and -high groups in cohorts 1 and 2. (J) Boxplots of TEAD IHC H-scores between NCOR1-low and -high groups in cohorts 1 and 2.

Epigenetic regulators affect gene expression via chromatin structure regulation, including histone protein modification, DNA methylation, TF binding, and chromatin accessibility^32^. With *NCOR1* amplification frequently observed (Figure 2C), we investigated epigenetic regulator gene alterations in cohort 1 (Figure 5A). *NCOR1* showed highest alteration frequency (35%), followed by *SMARCA4* (13%), *BRD4* (12%), and *CARM1* (12%). *BRD4*, *NSD1*, and *SMARCA4* alterations differed significantly between subtypes. *NCOR1* was amplified in over 30% of P2/P3 samples versus 14.2% in P1 (Figure 5B). *NCOR1* copy number correlated with NCOR1 protein level, with P3 showing a trend for highest expression. P2/3 of TCGA STLMS showed significantly higher *NCOR1* amplification frequency, resulting in highest *NCOR1* mRNA expression in P3 (Figure S5AB). As *NCOR1* was a top altered epigenomic regulator enriched in poor-outcome P3, we investigated the impact of high vs. low NCOR1 protein expression. We divided our cohort by median NCOR1 protein expression and performed a differential expression analysis with GSEA. GSEA revealed muscle organ development terms enriched in the NCOR1-high group, while immune and epithelial process terms were depleted (Figure 5E), suggesting NCOR1's role in muscle differentiation. Smooth muscle differentiation markers trended higher in the NCOR1-high group (Figure 5F), with TCGA STLMS data showing similar results (Figure S5C). The NCOR1-high group showed significantly higher stemness scores (Figures 5G, S5D) and PROM1 (CD133) and ALDH7A1 protein expression (Figure 5H). TCGA data showed higher *POU5F1* (Oct3/4), *PROM1* (CD133), and *ALCAM* mRNA in the *NCOR1*-high group (Figure S5E). Consistent with the Hippo pathway's role in stemness, we found YAP1/TEAD expression significantly higher in the NCOR1-high group (Figure 5IJ), confirmed by TCGA data showing YAP1/TAZ (encoded by WWTR1)/TEAD enrichment (Figure S5F). These findings suggest a role for NCOR in stemness maintenance and smooth muscle differentiation regulation.

### Enrichment of homologous recombination deficiency and nonhomologous end joining in poor-prognosis subtypes

**Figure 6.**
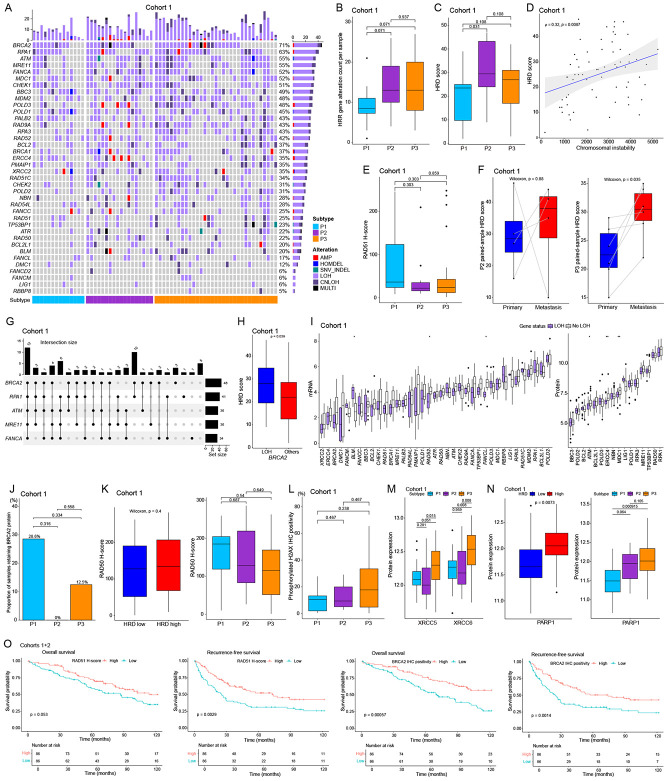
Homologous recombination repair pathway (A) Oncoprint of HRR pathway components showing frequent LOH events in cohort 1. (B) Boxplot of genomic alteration counts in the HRR pathway per sample shows high occurrence in P2/P3, but not statistically significant. (C) The HRD score is higher in P2/P3 compared to P1. (D) The HRD score positively correlates with CIN. (E) The RAD51 IHC scores are lower in P2/P3 compared to P1, which is concordant with the HRD scores of these subtypes. (F) Boxplot of HRD score comparison between paired primary and metastatic samples of P2 and P3. Metastatic samples of P3 show a significant increase. Although overall not statistically significant, 3 out of 4 samples of P2 show HRD score increases. (G) UpSet plot of thetop 5 altered genes. BRCA2 is the top altered gene with multiple different cooccurrence partners. (H) Boxplot of HRD scores between BRCA2 LOH and non-LOH groups showing significant increase of HRD scores in the LOH group. (I) Boxplots of mRNA and protein expression between LOH and non-LOH groups for each HRR gene. Among 37 HRR genes (*FANCD2* was excluded due to low LOH event number), 16 proteins were quantified in cohort 1 and are shown here. (J) Bar plot showing frequencies of BRCA2-retaining samples in each subtype. P2/P3 subtypes, especially P2, show decrease of BRCA2 retention compared to P1. (K) The RAD50 IHC score does not change significantly between HRD-low and HRD-high groups, but it shows a downward trend in P2/P3 compared to P1. (L) Boxplot of phosphorylated H2AX IHC scores by subtypes. (M) Boxplot of XRCC5/6 protein expression by subtypes. P3 shows significantly higher expression of XRCC5/6 compared to P1 or P2. (N) Boxplot of PARP1 protein expression between HRD-low and HRD-high groups by subtypes. (O) Kaplan-Meier analysis of overall and recurrence-free survival in combined cohorts 1 and 2, stratified by median values of RAD51 H-score and BRCA2 tumor cell positivity.

We observed high chromosomal instability (CIN) in P2/P3 (Figures 2B, S2B) with whole genome duplication and DNA repair enrichment in P3 (Figure 2H). The main repair pathway for DNA double strand breaks (DSB) is homologous recombination repair (HRR)^33^. Inactivation leads to genomic alterations, CIN, poor prognosis, and metastasis in tumors^34-36^. In cohort 1, we examined 64 HRR pathway genes from the TCGA sarcoma cohort^37^ and found 38 genes altered. Using allele specific copy number and variant calls, HRR genes showed frequent loss of heterozygosity (LOH) (Figure 6A). HRR gene events were enriched in P2/P3 (Figure 6B). The HRD (homologous recombination deficiency) score was highest in P2 (Figure 6C) and correlated with CIN (Figure 6D). The TCGA dataset confirmed these findings (Figure S6A-D). We additionally assessed RAD51 protein (a key recombinase for homologous recombination^38^) expression by IHC, finding a trend for decreased RAD51 staining in P2/P3 vs. P1 (Figure 6E), also supported by TCGA STLMS RPPA data (Figure S6E). Analysis of paired samples from the same patients showed increased HRD scores in metastatic sites vs, primary sites for P3 STLMS (Figure 6F), aligning with CIN-metastasis association^35,36^. We analyzed genomic alteration patterns of the top 5 altered HRR genes in STLMS (*BRCA2*, *RPA1*, *ATM*, *MRE11*, and *FANCA*) in cohort 1. The most frequent combination affected all 5 genes simultaneously (Figure 6G). For single or 2-gene combinations, *BRCA2* was predominant (total events = 17). *BRCA2* LOH tumors showed higher HRD scores than non-LOH tumors (Figure 6H). Assessment of LOH effects of the remaining 37 HRR genes on both mRNA and protein levels revealed decreased expression in LOH-positive STLMS (Figure 6I), a finding also supported by the external TCGA STLMS mRNA dataset (Figure S6F). TCGA STLMS RPPA protein data confirmed reduced BRCA2 protein in HRD-high and BRCA2-LOH groups (Figure S6G). BRCA2-loss by IHC was frequent in P2 (Figure 6J), which showed the highest HRD score among subtypes (Figure 6C). We assessed RAD50 expression in tumor tissue by IHC since RAD50 is a core protein of DSB recognition, a starting point of the HRR pathway. While HRD status alone showed no statistical difference in RAD50 expression in cohort 1, P2/P3 subtypes featured decreased RAD50 expression versus P1 (Figure 6K), a finding also supported by external TCGA data (Figure S6H). Assessment of phosphorylated H2AX (DNA double-strand break marker) showed a positivity range shift between subtypes (Figure 6L), suggesting impaired DSB recognition and repair in P2/P3 compared to P1. We also evaluated nonhomologous end joining (NHEJ), an alternative DSB repair process. We assessed both classical NHEJ driven by KU70/80 (coded by XRCC6/5) and alternative NHEJ driven by PARP1^39^. While XRCC5/6 increased in P3 compared to P1, P2 showed no significant difference to P1 (Figure 6M). PARP1 showed increased expression in the HRD-high group and the P3 subtype (Figure 6N), with P2 showing a close-to-significant increase, suggesting NHEJ pathway activation in P3 and possibly P2/P3. External TCGA data confirmed these results (Figure S6I). Using RAD51 and BRCA2 IHC protein expression data from cohorts 1 and 2, we found decreased expression of both proteins significantly associated with poor patient outcome (Figure 6O).

### Tumor immune microenvironment profiling reveals possible mechanisms of immune evasion

**Figure 7.**
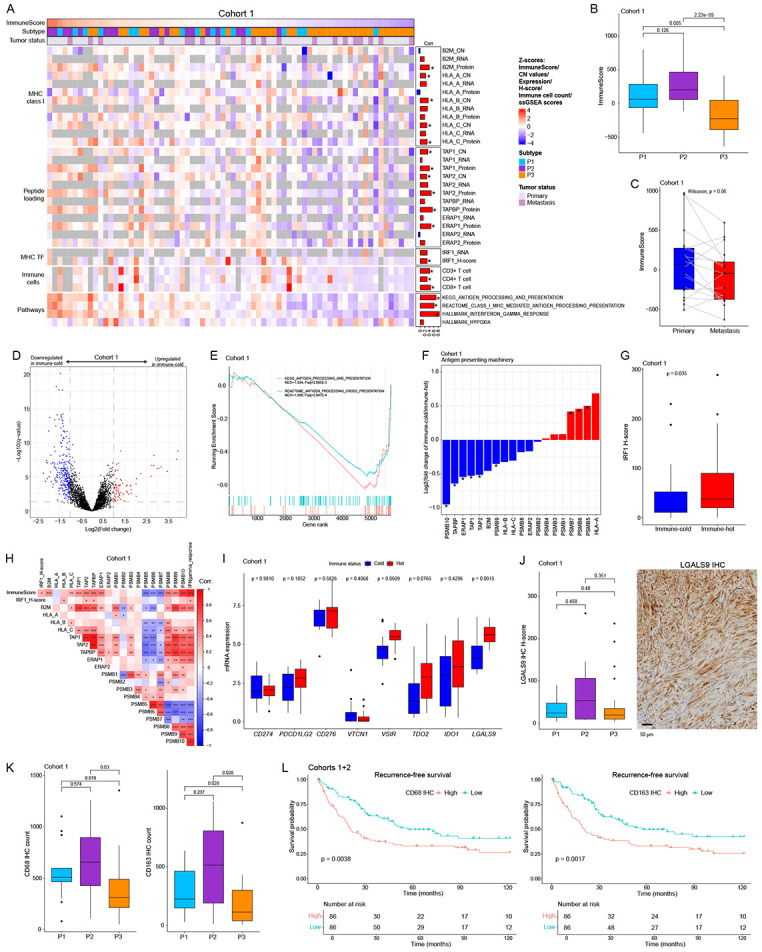
Tumor microenvironment profiling shows subtype differences and immune evasion characteristics in both immune-cold and immune-hot tumors (A) A heatmap of immune signatures including copy number status, mRNA, and protein level. In general, the ImmuneScore positively correlates with CD3 count, antigen presenting molecules, and immune-related pathways. Bars on the right side of the heatmap denote Spearman’s correlation coefficients between ImmuneScores and each gene/signature. (B) Boxplot showing the ImmuneScore by proteome subtypes for cohort 1. (C) Boxplot showing the ImmuneScore between paired primary and metastatic tumors. (D) Volcano plot showing differential protein expression results between immune-cold and immune-hot. Proteins with statistical significance (q<0.05) are shown in red (log2(fold change)>1) and blue (log2(fold change)<−1). The horizontal dashed line denotes q=0.05. (E) GSE plot of antigen presenting pathway-related KEGG and REACTOME terms, showing antigen presenting processes are significantly downregulated in immune-cold tumors compared to immune-hot tumors. (F) A bar plot of APM protein expression ratios between immune-cold and immune-hot in cohort 1. * denotes statistical significance in the differential expressional analysis shown in (D). (G) Boxplot of IRF1 protein expression between immune-hot and immune-cold tumors (H) A correlation plot (colored by Spearman correlation coefficient) including the ImmuneScore, IRF1, and antigen processing machinery protein expression. *, p<0.05; **, p<0.01; ***, p<0.001. IFNgamma_response scores are ssGSEA scores of the “Hallmark_Interferon_Gamma_Reponse” term. (I) Boxplot of immune-suppressive molecules between immune-cold and immune-hot tumors showing only TIM3 is significantly upregulated in immune-hot tumors vs. immune-cold tumors. (J) Boxplot of LGALS9 protein expression by subtypes (left side) and representative IHC image (right side). P2 shows a trend for higher expression of LGALS9 compared to P1 or P3. Bar, 50 μm. (K) Boxplots of CD68-positive or CD163 -sitive cell counts by subtypes. (L) Kaplan-Meier analyses of recurrence-free survival in combined cohorts 1 and 2, stratified by median values of CD68-positive or CD163-positive cell counts.

To elucidate cancer immune evasion mechanisms, we performed integrated proteogenomic analyses with IHC assessment of immune cell infiltration. Immune microenvironment status was determined using the ESTIMATE ImmuneScore^40^, which correlates with the CD3+ T-cell count by IHC (Figure 7A). The ImmuneScore correlates with antigen presenting machinery (APM) components, including MHC class I pathway members and IRF1 (Figure 7A). APM-related molecules showed good concordance between genomic copy number, mRNA, and protein expression (Figure 7A), indicating genomic alterations affect the immune microenvironment. The P3 subtype of STLMS showed a significantly reduced ImmuneScore (Figure 7B), a finding corroborated by external TCGA data (Figure S7AB). Paired sample analyses showed metastatic tumors had lower ImmuneScores (Figure 7C) and downregulated immune-related processes (Figure S7C). Comparing immune-cold vs. immune-hot tumors revealed 356 differentially expressed proteins, with 75 upregulated and 281 downregulated in immune-cold tumors (Figure 7D, Table S4). GSEA confirmed downregulation of antigen-presenting pathways in immune-cold tumors (Figure 7E), also supported by TCGA STLMS data (Figure S7D). Metastatic tumors showed similar downregulated antigen processing pathways (Figure S7E). ERAP1, TAP1/2, MHC class I complex molecules (HLA-B/C, B2M), and proteasome components were downregulated in immune-cold tumors (Figure 7F). IRF1, a major transcription regulator of MHC class I and APM molecules^41^, showed lower expression in immune-cold tumors (Figure 7G). We observed positive correlations between IRF1 and MHC class I molecules (HLA-B/C, B2M), though not statistically significant. IRF1 correlated positively with proteins in antigen processing (including immunoproteasome components), such as TAP1, TAP2, TAPBP, ERAP1, PSMB8, and PSMB9, while catalytic subunits of proteasome (PSMB5, PSMB7) were negatively correlated (Figure 7H), findings supported by TCGA transcriptome data (Figure S7F). IRF1 correlated with IFNγ-JAK-STAT pathway, which regulates its expression^42^, suggesting IFNγ signaling may have therapeutic potential against immune-cold STLMS^43-45^. Hypoxic environments may suppress host immune responses against tumors^46-48^. However, we found no significant correlation between hypoxia and the STLMS ImmunsScore (Figure 7A).

Next, we assessed the expression of tumor immune suppressors in immune-hot tumors, which may abrogate an immune response. Transcriptome analysis of cohort 1 found LGALS9 was upregulated in immune-hot tumors (Figure 7I). TCGA STLMS data showed high expression of immune suppressive molecules including LGALS9 (Figure S7G). As LGALS9 overlapped between the two cohorts, we evaluated its protein expression by IHC in cohorts 1 and 2. IHC confirmed tumor cells expressed LGALS9 with a trend for higher expression in the P2 (immune-hot) subtype (Figure 7J). Interestingly, LGALS9 protein expression was not detectable by IHC in benign smooth muscle, such as blood vessel walls or intestinal walls. The TCGA STLMS cohort showed a similar result (Figure S7H). Given LGALS9 upregulation in immune-hot tumors, we assessed tumor-associated macrophages via CD68 (pan-macrophage marker) and CD163 (M2 macrophage marker) IHC to evaluate their contribution to an immunosuppressive microenvironment. M2-type macrophages were enriched in the P2 subtype (Figure 7K). Survival analyses showed high CD68 or CD163 cell counts correlated with shorter recurrence-free survival (Figure 7L). These results suggest that the P2 subtype evades immune surveillance through LGALS9 expression and M2 macrophage recruitment, creating an immunosuppressive microenvironment (despite an immune-hot background) that is associated with poor clinical outcome.

**Figure 8.**
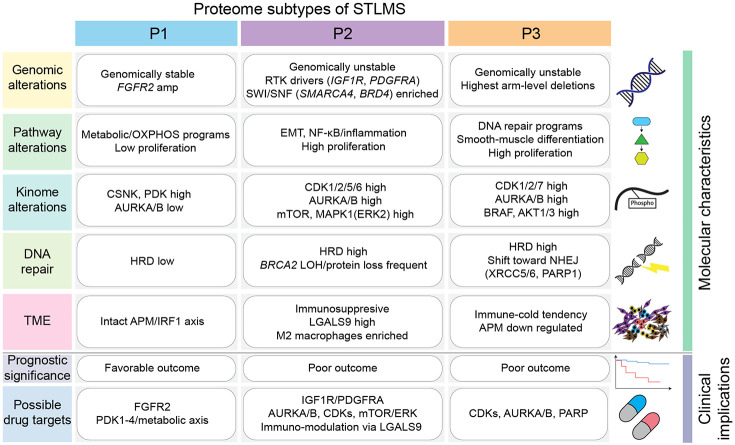
Integrated summary of proteome-defined STLMS subtypes Schematic overview of proteome-defined STLMS subtypes (P1-P3). Major genomic alterations, pathway alterations, kinome alterations, DNA repair alterations, tumor microenvironment features, prognostic trends, and hypothesis-generating therapeutic targets are summarized. APM, antigen-processing machinery; EMT, epithelial-mesenchymal transition; HRD, homologous recombination deficiency; LOH, loss of heterozygosity; NHEJ, non-homologous end-joining; TME, tumor microenvironment.

To concisely summarize the multi-omic findings, we generated an integrative schematic of the proteome-defined subtypes of STLMS (Figure 8). The figure summarizes, for each subtype, the characteristic genomic and pathway alterations, kinome alterations, DNA-repair features, tumor microenvironment, prognostic associations, and hypothesis-generating drug targets.

## Discussion

In this detailed proteogenomic analysis of STLMS, we identified three proteomic subtypes (P1, P2, and P3) with notable clinical and molecular differences (Figure 8). These subtypes are associated with patient outcomes, genomic alterations, signaling pathway activations, and immune microenvironment profiles, offering insights into STLMS heterogeneity and potential targeted therapies.

Unsupervised clustering of proteomic data identified the P2 subtype as having the worst prognosis with the shortest overall survival (OS) and recurrence-free survival (RFS). Both P2 and P3 subtypes were significantly enriched in metastatic behavior and higher-grade tumors, linking proteomic profiles to tumor aggressiveness. Validation using an external TCGA STLMS dataset confirmed the clinical relevance of our proteomic subtyping. The TCGA study identified only two subtypes (iClusters 1 and 2)^12^ but lacked comprehensive (phospho)proteomic data, focusing mainly on the AKT signaling pathway. Our integrated proteogenomic analyses, however, identified three molecular subtypes, providing a more detailed characterization that enhances understanding of STLMS tumor biology and heterogeneity.

Genomic analysis shows key differences in genomic instability between P1, P2, and P3 subtypes. High ploidy and whole-genome duplication in P2 and P3 indicate aggressive tumor phenotype, linked to rapid evolution and poor prognosis^49^. Enhanced chromosomal instability (CIN) in these subtypes may drive tumor heterogeneity and therapy resistance^50^. Higher alterations in oncogenic drivers like *IGF1R* and *PDGFRA* in P2 and P3 indicate activation of pathways of cell proliferation and survival^49^. Frequent alterations in SWI/SNF complex genes in P2, including *SMARCA4* and *BRD4*, suggest disrupted chromatin remodeling, causing aberrant gene expression and tumorigenesis^51^. The co-occurrence of *NCOR1* and *MAP2K4* gene amplifications in P2 and P3 is significant; NCOR1 regulates gene expression through chromatin remodeling, and MAP2K4 is vital in the MAPK/ERK pathway^49^. These amplifications contribute to tumor progression by maintaining cancer stemness and activating RTK pathways, crucial for cancer progression and metastasis^49^.

Copy number alterations (CNAs) correlated with mRNA and protein expression levels, particularly in genes linked to cell cycle regulation (Figure 3). The P3 subtype showed highest cell cycle activity and elevated AURKA expression, revealing potential therapeutic targets such as CDK1/2 and AURKA against aggressive STLMS subtypes. CRISPR/Cas9 gene perturbation experiments have supported the viability of these targets, offering promising avenues for targeted therapies^52,53^.

The P1 subtype shows low proliferation and metabolic process enrichment, suggesting slower-growing tumors with metabolic vulnerabilities. The P2 subtype exhibits EMT, NF-κB signaling, immune responses, and RAS pathways, indicating an aggressive, metastatic phenotype. The P3 subtype shows cell cycle activation and DNA repair dysregulation, suggesting highly proliferative tumors susceptible to therapies targeting cell cycle checkpoints or DNA repair deficiencies (Figures 2, 3, 6, 7). Phosphoproteomic analyses have revealed subtype-specific kinome programs corresponding to biological characteristics and therapeutic hypotheses. In subtype P1, kinase activity differed from P2 and P3, favoring metabolic regulation over proliferation. CSNK1A1 and the pyruvate dehydrogenase kinase axis (PDK1-PDK4) were selectively enriched, while AURKA/B activity was diminished (Figure 4C-D). This aligns with oxidative-metabolic pathway enrichment and low proliferation in P1 and *FGFR2* alterations. These results suggest a metabolically reprogrammed, growth factor-dependent, yet cell cycle-quiescent state may be targetable with FGFR2/PDK-axis strategies (Figures 2H,4A, and 8). P2 showed an RTK-RAS/ERK-mTOR cell cycle signature. *IGF1R*/*PDGFRA* alterations were enriched, and CDK1/2/5/6, AURKB, mTOR, and MAPK1(ERK2) were highly scored, with downstream S6K/RSK families (Figure 4A-D). These signals match the EMT phenotype and poor outcomes of P2, supporting CDK1/2 or AURKB blockade with mTOR/ERK pathway inhibition (Figures 2H and 4C-D) as therapeutic targets. Subtype P3 showed a proliferative mitotic/replication kinome with CDK2/7, AURKB, ROCK1, and BRAF activity, and elevated AKT1/3 compared to P1/P2 (Figure 4C-D). Together with P3’s high cell-cycle scores and AURKA protein, these data suggest sensitivity to CDK/Aurora inhibitors, while ROCK1 and BRAF enrichment implies actomyosin and MAPK dependencies (Figures 3D-H and 4C-D). AURKB activity was elevated in P2/P3 versus P1, validated by IHC, identifying AURKB as a cross-subtype mitotic driver (Figure 4E-F). AURKB-T232 motif analysis suggested upstream mitotic and checkpoint kinase activation in P2/P3, supporting AURKB-targeted therapy (Figure 4G). These kinome data define P1 as PDK-activated with metabolic/FGFR2 bias, P2 as RTK-ERK-mTOR/CDK-driven, and P3 as CDK/Aurora-centered with ROCK/MAPK inputs (Figures 4 and 8).

Transcriptional regulatory network analysis identified multiple transcription factors for each subtype (Figure 2I). These findings highlight the complexity of STLMS biology and importance of understanding subtype-specific regulatory networks for targeted therapies. Future studies should focus on validating these transcription factors as therapeutic targets and exploring their regulatory network interactions. The P2 subtype showed elevated activity of oncogenic regulators, including MYC. MYC is an oncogene that drives cell proliferation, growth, and metabolism. Its activation links to aggressive tumor phenotypes and poor prognosis^56^, aligning with P2's worse outcome (Figure 1B). The P3 subtype showed enrichment for cell cycle-related transcription factors, including E2Fs, DNMT1, and PAX6, suggesting epigenetic regulation in its pathogenesis.

NCOR1 expression in our cohort was associated with both increased smooth-muscle marker expression and higher expression of progenitor-like stemness markers (PROM1 (CD133) and ALDH7A1) (Figure 5). NCOR1, as a chromatin corepressor interacting with HDAC3, can reshape transcriptional programs to favor a lineage-restricted progenitor state that co-expresses differentiation markers while retaining self-renewal properties, which is a behavior seen in muscle physiology and chromatin regulation. Additionally, the increased activity of YAP1/TAZ-TEAD in tumors with high NCOR1 levels suggests a potential collaborative pathway, as YAP/TEAD signaling encourages the proliferation of muscle progenitors and supports regenerative processes. These combined characteristics might contribute to a differentiated yet progenitor-like phenotype that is resistant to therapy in STLMS. To validate this hypothesis, functional disruption and chromatin analysis will be necessary^57^.

Our observations showed that HRD is common in both P2 and P3 subtypes, alongside activation of NHEJ pathways (Figure 6)^58^. Elevated HRD scores correlated with increased chromosomal instability. Loss of heterozygosity in HRR genes, notably *BRCA2*, was linked to reduced HRR protein expression and unrepaired DNA double-strand breaks, as shown by higher phosphorylated H2AX levels (Figure 6L). PARP1 upregulation suggests that PARP inhibitors may be effective therapeutic agents in HRD-high STLMS (Figure 6N). HRD, a hallmark of genomic instability, is characterized by inability to repair DNA double-strand breaks through homologous recombination^59^. This deficiency leads to reliance on error-prone repair mechanisms like NHEJ, resulting in chromosomal rearrangements and mutations. In sarcomas, particularly uterine leiomyosarcoma, HRD is associated with aggressive tumor behavior and poor prognosis^60^. Identification of HRD in two of our proteomic subtypes of STLMS points to the potential for targeted therapies against STLMS exploiting this vulnerability, such as PARP inhibitors.

Immune profiling analysis revealed that the P3 subtype and metastatic tumors exhibit lower ImmuneScores and downregulation of antigen-presenting machinery (APM) components, contributing to an immunosuppressive tumor microenvironment. Reduced expression of IRF1 and APM molecules in immunosuppressed tumors may facilitate immune evasion mechanisms. Conversely, immune-hot tumors demonstrated concomitant upregulation of suppressive immune checkpoint molecules, such as LGALS9, and M2 macrophage recruitment. These results suggest that combinatory immunomodulatory therapies, such as LGALS9 or PD-L1 inhibition^61^, could be strategically tailored to the immune profile and proteomic subtype of STLMS.

Our study presents a large, systematic proteogenomic analysis of STLMS that also includes a phosphoproteomic characterization of kinome alterations. By identifying subtype-specific genomic alterations and pathway activations, the study establishes a foundational molecular basis for precision diagnostics and therapeutic interventions. Aggressive STLMS subtypes may benefit from therapies targeting CDK1/2 and AURKA or from exploiting HRD with PARP inhibitors. Patients with immune-hot tumors expressing LGALS9 may respond to immune checkpoint inhibitors. These findings highlight clinically relevant heterogeneity within STLMS and suggest that future precision medicine testing should incorporate proteogenomic or surrogate assays to assign molecular subtypes and guide risk stratification and treatment selection. Our comprehensive proteogenomic dataset of primary and metastatic STLMS provides a valuable resource for further validation of these observations and for the development of practical assays to implement subtype–based management in routine care for patients with STLMS.

## Materials and Methods

### Clinical specimens and pathological data

We examined tissues from two cohorts of STLMS patients. Cohort 1 consists of 72 primary and metastatic STLMS tissues from 54 patients, including 18 primary-metastasis-paired samples. Cohort 2 consists of 222 surgically resected STLMS samples. The study was approved by the Institutional Review Board (MSKCC #16-1683, BIDMC #2023P000933). Clinical data, such as patient demographics, treatment history, recurrence status, clinical targeted MSK-IMPACT sequencing results, or histologic grade were retrieved from medical records in an anonymized fashion. Tumor content ratios for all samples were verified by board-certified pathologists. Clinicopathologic features and prognosis information, including gender, age, pathological TNM stage, anatomic site, are summarized in Table S1.

### Targeted cancer gene sequencing

MSK-IMPACT targeted sequencing was conducted as previously outlined^62^. In summary, genomic DNA was extracted from both tumor and adjacent normal tissues using the DNeasy Tissue kit and the EZ1 Advanced XL system (Qiagen). The extracted DNA was fragmented with the Covaris E200 device. Custom DNA probes were crafted for targeted sequencing of all exons and selected introns across 505 genes. These probes were synthesized using the NimbleGen SeqCap EZ library custom oligo system and were biotinylated. Sequencing libraries were prepared following the KAPA HTP protocol (Kapa Biosystems) and the Biomek FX system (Beckman Coulter), and sequencing was performed on the Illumina HiSeq 2500 to achieve high, uniform coverage (>500x median coverage). Demultiplexed fastq files underwent trimming with Trim Galore v0.2.5mod (https://github.com/FelixKrueger/TrimGalore) to remove adapters and short reads. These files were then aligned to the human reference genome (GRCh37) using BWA-MEM v0.7.5a (with arguments -M -t 6)^63^, and Picard Tools v2.9 (https://github.com/broadinstitute/picard) was used. AddOrReplaceReadGroups was applied to annotate read groups, and MarkDuplicates was used to identify PCR duplicates. Genomic regions were pinpointed using FindCoveredIntervals from the GATK Tool Kit v3.3-0^64^and underwent indel realignment with Assembly Based ReAligner (ABRA) v2.12^65^. GATK BaseRecalibrator was employed to identify systematic errors in base quality scores. Variant calling was executed in paired tumor/normal mode using MuTect v1.1.4^66^for single nucleotide variants (SNV). Pindel v0.2.5a7^67^was utilized for small insertions and deletions (indels). Vardict v1.5.1^68^served as the variant caller to report both SNVs and indels. The vcfs generated by MuTect, Vardict, and Pindel were subsequently combined. Additionally, copy-number variants, including chromosomal instability (CIS) and whole-genome doubling (WGD), were identified using FACETS^69^. The resulting variants were annotated using vcf2maf v1.6.14 (https://github.com/mskcc/vcf2maf), which employs Ensembl’s Variant Effect Predictor v86. In conclusion, we successfully conducted MSK-IMPACT sequencing on 69 out of 72 samples in cohort 1 (LM_010P, LM_020P, LM_043P lacked sufficient DNA for sequencing). Amplifications and deep deletions were identified from the GISTIC result table named “allthresholded.bygenes.txt” (part of the GISTIC output used to determine the copy number status of each gene in each sample). Genes with a value of +2 were classified as amplifications, while those with a value of −2 were considered deep deletions. Sequencing results are detailed in Table S5.

### Allele-specific copynumber analysis

FACETS analyses were conducted to ascertain genome-wide allele-specific and absolute DNA copy numbers for all samples (FACETS version 0.5.6)^69^. Allele-specific copy number data were utilized to estimate tumor purity and ploidy. Prior to further analyses, total copy number log ratios were adjusted for ploidy and purity. Tumors exhibiting whole-genome doubling (WGD) were identified as those in which more than 50% of the autosomal genome possessed a major copy number ≥2, where the major copy number is defined as the number of copies of the most prevalent allele present in the sample^70^. Loss of heterozygosity (LOH) was defined as the loss of one allele in cohort 1. For the TCGA STLMS cohort, allele-specific copy number data were obtained via the R TCGAbiolink package^71^, and LOH was defined using the same criteria.

### HRD score calculation

The scarHRD R package was utilized to calculate the HRD score of our cohort 1 samples^72^. This metric was derived from the simple addition of three types of genetic alterations: loss of heterozygosity (LOH), large-scale transitions (LST), and telomeric allelic imbalance (TAI) based on allele-specific copy number data. LOH refers to the elimination of genomic segments exceeding 15 Mb in size, excluding entire chromosomes. LST encompasses chromosomal breakages between adjacent regions of at least 10 Mb, with breaks occurring no more than 3 Mb apart. TAI quantifies the disparities in allele sequence contributions within the telomeric regions of chromosomes. For the TCGA cohort, we downloaded precalculated HRD score from the prior study^73^.

### Chromosomal instability inference

Chromosomal instability (CIN) scores of our cohort 1 and the TCGA STLMS data was computed on the allele-specific copy number segments by adding the number of gains (CNV >2), losses (CNV <2), and loss of heterozygosity per sample based on data from the prior study^37^.

### Recurrent somatic copy number alteration detection

Recurrent somatic copy number alterations (SCNAs) were identified using the Genomic Identification of Significant Targets in Cancer (GISTIC, version 2.0.23)^74^to ascertain which SCNA regions exhibited amplification or deletion at a frequency greater than expected by chance, with a q value threshold of 0.05. The analysis employed the following parameters: Amplification Threshold = 0.3, Deletion Threshold = 0.3, Cap Values = 1.5, Confidence Level = 0.99, Join Segment Size = 4, Arm Level Peel Off = 1, and Sample Normalization Method = mean. Default values were applied for all other parameters.

### RNA sequencing

For each sample, 20-30 mg of frozen tissue was homogenized in 1 mL of TRIzol Reagent (ThermoFisher catalog # 15596018), followed by phase separation induced with 200 μL of chloroform. RNA was extracted from 350 μL of the aqueous phase utilizing the miRNeasy Mini Kit (Qiagen catalog # 217004) on the QIAcube Connect (Qiagen) in accordance with the manufacturer’s protocol. Samples were eluted in 34 μL of RNase-free water. Subsequent to RiboGreen quantification and quality control via the Agilent BioAnalyzer, 500 ng of total RNA with RIN values ranging from 5.9 to 9.9 underwent polyA selection and TruSeq library preparation as per the instructions provided by Illumina (TruSeq Stranded mRNA LT Kit, catalog # RS-122-2102) with 8 cycles of PCR. Samples were barcoded and sequenced on a NovaSeq 6000 in a PE100 run, employing the NovaSeq 6000 S1 Reagent Kit (200 Cycles) (Illumina). Read quality was evaluated using FastQC. Trimmed raw reads were aligned to the human genome version hg19 using STAR (v2.7.5a)^75^with default parameters, and gene annotation was incorporated using Gencode v36, followed by the calculation of gene-level count values and TPM values via the RSEM tool (version 1.3.1)^76^.

### Tissue proteome extraction

Aliquots of 5 mg of frozen tissue were lysed using a lysis buffer composed of 8 M urea and 200 mM EPPS (4-(2-Hydroxyethyl)-1-piperazinepropanesulfonic acid), at pH 8.5, supplemented with protease inhibitor (complete mini EDTA-free, Roche) and phosphatase inhibitors (cocktails 2 and 3, Sigma). This was followed by homogenization using a Bead Ruptor. Subsequently, the tissue mixture underwent sonication through 12 cycles of 1-minute sonication at 120 W power (FB120, Fisher Scientific), with intermittent cooling. Post-centrifugation at 18,000 g for 10 minutes at 4 °C, the supernatant containing all soluble proteins was collected. Protein concentrations were quantified using BCA assays (Pierce). Pooled protein samples were prepared and stored, with equal amounts of all samples combined to assess MS run quality.

### Liquid chromatography- mass spectrometry (LC-MS) proteomic analysis

Protein samples were labeled utilizing the 16-plex TMT chemical labeling reagent (Thermo Fisher Scientific), followed by MultiNotch MS3 LC–MS analysis employing an Orbitrap Fusion MS^77^. In brief, 300 μg of proteins were reduced with 5 mM TCEP (tris(2-carboxyethyl) phosphine hydrochloride), alkylated with 10 mM IAA (iodoacetamide), and quenched with 10 mM DTT (dithiothreitol). The samples were subsequently diluted and precipitated using chloroform-methanol^78^. The resulting pellets were resuspended in 50 μL of 200 mM EPPS buffer and digested with Lys-C and trypsin at 37 °C overnight. Anhydrous acetonitrile was added to achieve a final volume of 30%. TMTPro (16-plex) reagents were introduced to the peptides at a 2.8:1 (TMT reagent-to-peptide) ratio and incubated for 1 hour at room temperature. A label ratio check was conducted to determine mixing ratios, labeling efficiency, and missed cleavages by pooling 1 μL from each sample. Based on these results, samples were mixed to achieve equal intensity across TMT channels. The samples were dried to remove acetonitrile, desalted using C18 solid-phase extraction (SPE) Sep-Pak (Waters), and vacuum-centrifuged. Five percent of the desalted samples were reserved for global proteome analysis, while 95% were utilized for phosphopeptide enrichment. Dried peptides intended for global proteomics were fractionated using the Pierce High pH Reversed-Phase Peptide Fractionation Kit (ThermoFisher catalog # 84868) into nine fractions per the modified manufacturer's protocol. The fractions were vacuum-centrifuged and then reconstituted in 1% ACN/0.1% FA for LC–MS analysis. For optimal phosphopeptide enrichment, phosphopeptides were collected using the High-Select TiO2 Phosphopeptide Enrichment Kit (Thermo Fisher) and from the stored flow-through of Fe-NTA using the Pierce High-Select Fe-NTA Phosphopeptide Enrichment Kit (Thermo Fisher), and both were combined. Phosphopeptide samples were fractionated into nine fractions utilizing the Pierce High pH Reversed-Phase Peptide Fractionation Kit. The fractions underwent analysis via LC-MS/MS employing a nanoAQUITY UPLC (Waters) with a 50 cm (75 μm inner diameter) EASY-Spray column (PepMap RSLC, C18, 2 μm, 100 Å) maintained at 60 °C, in conjunction with an Orbitrap Fusion Lumos Tribrid Mass Spectrometer (Thermo Fisher). Peptide separation was conducted at a flow rate of 300 nL/min using a linear gradient of 1-35% acetonitrile (0.1% FA) in water (0.1% FA) over a duration of four hours, analyzed in SPS-MS3 (for global proteome) and neutral loss-triggered MS3 (phosphopeptide) modes. For global proteome analysis, MS1 scans were performed within a range of 375–1,500 m/z, with a resolution of 120 K, an AGC target of 4×1e5, and a maximum IT of 50 ms. MS2 scans were conducted on MS1 scans of charges 2-7 using 0.7 m/z isolation, collision-induced dissociation at 32%, turbo scan, and a maximum IT of 50 ms. MS3 scans employed specific precursor selection (SPS) of 10 isolation notches, an m/z range of 100–1,000, 45% CE, 50 K resolution, and an AGC target of 1e5. For phosphopeptide analysis, MS1 scans were conducted within a range of 380-1,400 m/z, with a resolution of 120 K, an AGC target of 4×1e5, and a maximum IT of 50 ms. MS2 scans were performed on MS1 scans of charges 2-7 using 0.7 m/z isolation, collision-induced dissociation at 35%, turbo scan, and a maximum IT of 50 ms. Neutral loss-triggered MS3 scans utilized a range of 100–1,000 m/z, 38% CE, 60 K resolution, an AGC target of 1e5, and a maximum IT of 118 ms. Targeted loss masses of 97.9763 and 79.9658 were employed.

### TMT data analysis

Raw data files were processed utilizing Proteome Discoverer (PD) version 2.4.1.15 (Thermo Scientific). In each of the TMT experiments, raw files from all fractions were consolidated and analyzed using the SEQUEST HT search engine with a Homo sapiens UniProt protein database, downloaded on 2019/12/13 (92,249 entries). Oxidation (M), phosphorylation (STY), and deamidation (NQ) were designated as variable modifications, whereas cysteine carbamidomethylation, TMT 16plex (K), and TMT 16plex (N-terminus) were specified as fixed modifications. The precursor and fragment mass tolerances were set at 10 ppm and 0.6 Da, respectively. A maximum of two missed trypsin cleavages were allowed. Searches employed a reversed sequence decoy strategy to control the peptide false discovery rate (FDR), with a 1% FDR established as the identification threshold. Phosphosite localization was determined using the PD IMP-ptmRS node. The raw abundance of protein, phosphoprotein, and phosphopeptide levels was normalized by sample loading normalization of each TMT channel after filtering out protein/peptide assignments with valid values for less than 30% of samples and K-Nearest Neighbors (KNN) imputation. Internal reference scaling based on pooled samples assigned for each TMT plex was conducted across the entire cohort to eliminate batch effects, followed by TMM normalization for median centralization between TMT batches^79,80^. Differential expression analyses were performed using edgeR^81^.

### Unsupervised clustering of proteomic data

We used non-negative matrix factorization (NMF)-based clustering of global protein and phosphoprotein expression data. In brief, we constructed a merged expression matrix of normalized global protein and phosphoprotein data and selected the top 500 (phospho)proteins with highest mean absolute deviations for unsupervised clustering to avoid noisy (phospho)proteins in the data. The resulting matrix was then subjected to NMF analysis leveraging the NMF R package^82^. To determine the optimal factorization rank *k* (number of clusters) for the matrix, we tested a range of clusters between *k* = 2-7. For each *k*, we factorized matrix *V* using 1,000 iterations with random initializations of W and H. To determine the optimal factorization rank, we calculated two metrics for each k: (1) cophenetic correlation coefficient (measuring how well the intrinsic structure of the data was recapitulated after clustering) and (2) silhouette score (indicating how well each sample lies within its cluster compared to other clusters^83^) (Figure S1CD). These metrics indicate the reproducibility of the clustering across 1,000 iterations. The cophenetic correlation coefficients for *k* = 2 and 3 were >0.95 and showed a large drop at *k* = 4. The silhouette score at *k* = 2, 3 showed relatively higher scores than that at *k* = 4. Based on these results and census heatmaps for each *k*, we chose *k* = 3 as the optimal clustering number with sufficient samples in each class for downstream analysis.

### Gene set enrichment analysis (GSEA)

Fold change values for gene expression between specified groups were calculated using edgeR. We employed clusterProfiler (v2.1.2)^84^with a ranked gene list ordered by fold change value. Enrichment tests were conducted for GOBP, Hallmark, KEGG, and Reactome gene sets obtained from the Molecular Signatures Database (MSigDB v7.4). For the functional characterization of each sample via single sample GSEA, we calculated normalized enrichment scores (NES) of cancer-relevant gene sets by projecting the matrix of normalized expression data onto GOBP, Hallmark, KEGG, and Reactome pathway gene sets. This was done using the ssGSEA implementation available at https://github.com/broadinstitute/ssGSEA2.0 with the following parameters: sample.norm.type = “log”, weight = 0.75, statistic = “area.under.RES”, output.score.type = “NES”, nperm = 1000, min.overlap = 3, correl.type = “z.score”. To elucidate gene set enrichment in each sample, we applied single sample GSEA on normalized expression data^85^and calculated the normalized enrichment score (NES) for MSigDB terms.

### Over-representation analysis (ORA)

ORA was performed to identify biological pathways and functional categories enriched in the gene set of interest by using clusterProfiler (v2.1.2)^84^. Enrichment was calculated using a hypergeometric test against the defined background, and p-values were adjusted for multiple comparisons using the Benjamini-Hochberg method.

### Posttranslational modification signature enrichment analysis (PTM-SEA)

To infer kinase activity based on phophosite-specific quantification data, we performed PTM-SEA (https://github.com/broadinstitute/ssGSEA2.0), an adaptation of ssGSEA, that analyzes site-specific signatures by scoring PTMsigDB bi-directional signature sets. PTMsigDB, sourced from over 2,500 publications, contains modification site-specific signatures linked to perturbations, kinase activities, and signaling pathways. Unlike other pathway databases, PTMsigDB annotates each PTM site with its directional change upon specific perturbations or signaling events, improving PTM-SEA's scoring. PTMsigDB’s main source is the PhosphoSitePlus database^86^. The PTM-SEA requires two main inputs: a site-centric data matrix, denoted as m and formatted in GCT v1.3, along with the PTM signatures database. In matrix m, each row corresponds to a distinct phosphorylation site that is precisely localized to a particular amino acid residue, with the columns indicating the measured abundances across different samples. When multiple phosphorylation sites are identified on the same peptide, they are transformed into individual site-specific entities for each site.

### Kinase enrichment analysis between subtypes

To assess enrichment of activated kinases and phosphatases between groups based on functional information such as known protein-protein interactions or kinase-substrate annotations, we input phosphopeptide abundance change values (log2(fold change)) between proteome subtypes into the RoKAI application (v0.8.3)^31^ and obtained kinase activity scores at proteome subtype level. Default parameters were used.

### Transcriptional regulatory network analysis

To deduce the transcriptional regulatory activity of STLMS based on proteome subtype, we utilized the RTN R package^87-89^, which examines the link between specific transcription factors (TFs) or gene regulators and all possible targets, as well as the enrichment status of a network using proteome data. In summary, we employed normalized transcriptome data (n=27) and Lambert’s human TF list^90^(1,612 TFs) to initially build a potential transcriptional regulatory network (TRN). We then eliminated non-significant associations through permutation analysis (permutation n=1000) and bootstrapping, followed by the ARACNe algorithm^91^ to preserve direct TF-target interactions, thereby removing redundant indirect interactions. The refined TRN dataset includes the regulator gene name, its target gene name, and adjusted p-values as a confidence measure. For enrichment analyses over a list of regulons in the refined TRN, we first conducted a Master Regulator Analysis (MRA)^92^over a list of regulons, with adjustments for multiple hypothesis testing. The MRA evaluates the overlap between each regulon and the significantly dysregulated genes between subtypes, providing adjusted p-values, which resulted in 600 confident regulons (adjusted p-values <0.05) for further analyses. We then determined these regulons’ enrichment scores between proteome subtypes using transcriptome expressional fold change values and the GSEA-2T (two-tailed GSEA analysis) function from the RTN R package. To identify similarities and differences in regulatory programs among samples in our cohort, we selected 60 statistically robust regulons (adjusted p-values <1E-8), calculated the activity score of each regulon in each sample, and visualized the data as a heatmap. Additionally, we incorporated CRISPR perturbation scores (sourced from the Cancer Dependency Map (DepMap) Project^93^) of 36 sarcoma cell lines and included this data in the heatmap.

### Proteome subtype inference of TCGA STLMS samples

To externally validate differences found in cohort 1 between proteome subtypes (P1, P2, and P3) by using the TCGA STLMS transcriptome dataset, we constructed a random forest classifier of our proteome subtypes based on transcriptome data of cohort 1 (n=27). To select informative genes for model construction, we first conducted differential expression analyses between subtypes and selected only differentially expressed genes (FDR <0.01, absolute value of fold change >2) between subtypes (668 genes). Next, we divided normalized expression data of selected genes into training (n=19) and test (n=8) datasets, followed by random forest model training with 10-fold cross-validation using the tidymodel R package^94^. We applied the trained classifier to the test dataset and got a 1.0 F-means value (which means all sample subtypes were correctly predicted). We then applied the classifier to the TCGA STLMS transcriptome dataset (n=53) and inferred proteomic subtypes in the dataset. TCGA sample IDs and inferred proteomic subtypes are shown in Table S6.

### Assessment of immune status

ImmuneScores were derived from the normalized protein expression matrix of 72 samples utilizing the ESTIMATE (Estimation of STromal and Immune cells in MAlignant Tumor tissues using Expression data) package in R^40^. To evaluate immune cell infiltration, we conducted IHC with immune cell markers (CD3, CD4, CD8, CD68, CD163) and tallied the number of positive cells for each marker across 3 TMA cores per sample. Subsequently, we computed the average number of positive cells per core for each sample for further analysis.

### Tissue microarray (TMA) construction

TMAs were created from FFPE blocks of 67 STLMS tissues in cohort 1 and 222 STLMS tissues in cohort 2. Three 1-mm cores were extracted from each tissue paraffin block and placed into tissue array blocks using a TMA arrayer (3DHistech). Tumor and normal regions were chosen after a thorough examination of individual histologic slides and electronic image-based selection of target areas for coring.

### Immunohistochemistry (IHC)

Four-μm sections were cut from FFPE tissue blocks. Paraffin was removed with xylene, and antigens were retrieval by heat-mediated epitope retrieval. Tissue sections were stained using a Leica BOND-MAX IHC autostainer. The antibodies used were anti-CD3 (Leica Microsystems, NCL-L-CD3-565, 1/200 dilution), anti-CD4 (Sigma, 104R-15, 1/100 dilution), anti-CD8 (Dako, M7103, 1/1500 dilution), anti-CD68 (Dako, M0876, 1/200 dilution), anti-CD163 (Leica Microsystems, NCL-CD163, 1/250 dilution), anti-MITF (Abcam, ab303530, 1/100 dilution), anti-MYC (Abcam, ab32072, 1/200 dilution), anti-PAX6 (Abcam, ab195045, 1/100 dilution), anti-AURKA (Cell Signaling Technology, #91590, 1/100 dilution), anti-AURKB (Abcam, ab45145, 1/100 dilution), anti-CDK1 (Abcam, ab133327, 1/250 dilution), anti-CDK2 (Cell Signaling Technology, #18048, 1/200 dilution), anti-active YAP1 (Abcam, ab205270, 1/20,000, dilution), anti-TEAD (Abcam, ab97460, 1/400 dilution), anti-NCOR1 (Invitrogen, PA1-844A, 1/1000 dilution), anti-RAD51 (Abcam, ab133534, 1/100 dilution), anti-BRCA2 (Sigma, HPA026815, 1/50 dilution), anti-RAD50 (Cell Signaling Technology, #61915, 1/4000 dilution), anti-phosphorylated H2AX (Cell Signaling Technology, #9718, 1/200 dilution), anti-Ki67 (Biocare, PRM325, 1/100 dilution), anti-IRF1 (Abcam, ab243895, 1/100 dilution), anti-LGALS9 (Abcam, ab227046, 1/200 dilution). For MYC, MITF, CDK1, CDK2, NCOR1, YAP1, RAD50, RAD51, PAX6, IRF1, and LGALS9 IHC, The staining intensity of each tumor cell was rated on a scale from 0 to 3+, and the average was taken from three separate tissue cores for each case. To determine the total weighted IHC score (IHC H-score, theoretically ranging from 0 to 300) for a sample, the expression intensity of individual tumor regions (rated 0-3+) was multiplied by their respective contributions (0-100%) to the overall tumor area, and these values were then summed. Two pathologists independently evaluated all tissue samples. If there were any discrepancies, the cases were re-evaluated until a consensus score was achieved. For Ki67, AURKA/B, TEAD, BRCA2, and phosphorylated H2AX IHC, we assessed the fraction of positive tumor cells. BRCA2 loss was defined as less than 50% positivity.

### External datasets

The TCGA STLMS dataset (53 samples) including clinical information, transcriptome data (count data, TPM), and gene alterations with allele-specific copy number data (data for 1 sample was not available) was obtained via the TCGAbiolink R package (version 2.30.0)^71^. Protein expression data from reverse phase protein array (RPPA) experiments of the TCGA cohort was downloaded from MD Anderson Cancer Center database (https://tcpa.drbioright.org/rppa500/main.html).

## Supplementary Material

1

Table S1. Clinicopathological characteristics of patients in cohort 1, related to Figures 1-7

Table S2. Proteome and phosphoproteome expression data of cohort 1, related to Figures 1-7

Table S3. Differential protein expression analysis between subtypes, related to Figure 2

Table S4. Differential protein expression analysis between immune-cold and immune-hot samples, related to Figure 7

Table S5. MSK-IMPACT summary with GISTIC 2.0 results of cohort 1, related to Figure 2

Table S6. Clinicopathological attributes and inferred proteome subtypes of the TCGA STLMS cohort, related to Figures S1-S7

Document S1. Figures S1-S7

## Abbreviations (there are a lot more missing)

STLMS: soft tissue leiomyosarcoma
IHC: immunohistochemistry
SNV: single nucleotide variant
FFPE: formalin fixed paraffin embedded
GSEA: gene set enrichment analysis
PTM-SEA: posttranslational modification signature enrichment analysis
CIN: chromosomal instability
CNA: copy number alteration
DSB: DNA double-strand break
EMT: epithelial–mesenchymal transition
FDR: false discovery rate
APM: antigen-presenting machinery
