# Supplementary material for: Proteogenomic profiling of soft tissue leiomyosarcoma reveals distinct molecular subtypes with divergent outcomes and therapeutic vulnerabilities
Source: bioRxiv. 2025 Nov 23:2025.11.19.689365. Preprint. [Version 1] doi: 10.1101/2025.11.19.689365 (PMC12667925; doi:10.1101/2025.11.19.689365)
Supplement: 1 [file NIHPP2025.11.19.689365v1-supplement-1.pdf]

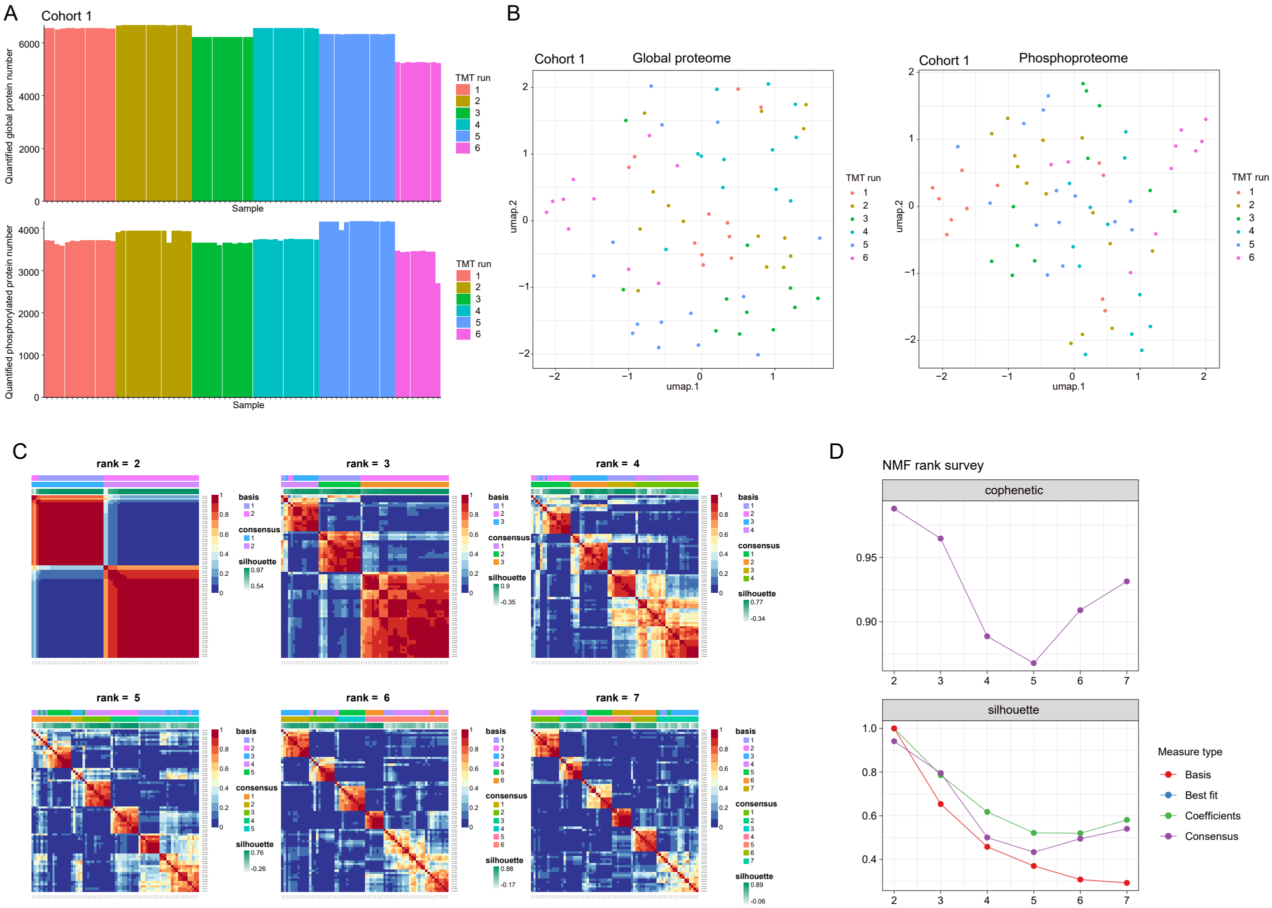

E

TCGA iCluster LMS subtypes	Inferred proteome subtype		
	P1	P2	P3
iCluster1	0	1	27
iCluster2	12	5	8

Fisher's exact test p=1.6E-7

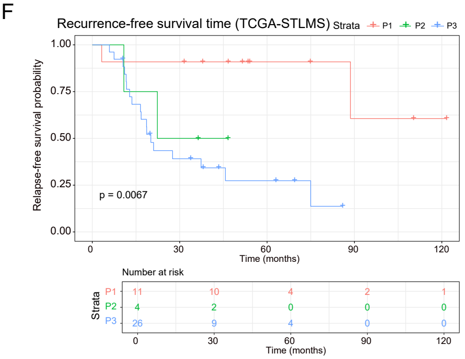

Figure S1. Evaluation of proteome data quality, batch effects, and robust subtype inference, related to Figure 1

(A) Bar plots showing the number of quantified proteins per sample for (top) global proteome and (bottom) phosphoproteome datasets. Samples are grouped and color-coded by TMT plex MS runs (1-6). The relatively consistent number of quantified proteins across samples and batches indicates robust and comparable proteome/phosphoproteome coverage across the TMT experiments.

(B) UMAP plots of (left) global proteome and (right) phosphoproteome profiles, colored by TMT plex MS runs (n=6). Each point represents a sample. Samples are well intermixed across TMT batches, indicating no detectable batch effect in both global proteome and phosphoproteome datasets.

(C) Consensus heatmaps for non-negative matrix factorization (NMF) clustering at ranks k=2-7, showing clustering stability across different cluster numbers. Each heatmap illustrates consensus values between sample pairs, with color bars indicating sample assignments in basis and consensus groupings. Silhouette width values are shown for each rank, reflecting clustering quality.

(D) Summary plots of NMF rank survey metrics: cophenetic coefficient (top) and silhouette width (bottom) across ranks. Rank 3 shows a relatively high silhouette score and a drop in cophenetic coefficient, suggesting it as the optimal cluster number balancing stability and separation.

(E) Association between proteome-defined subtypes (P1-P3) and TCGA iCluster LMS subtypes (iCluster1 and iCluster2) in the TCGA STLMS cohort. The distribution shows significant concordance between iCluster1 and subtype P3, and iCluster2 and subtypes P1/P2 (Fisher's exact test, p=1.6×10E-7).

(F) Kaplan-Meier analysis of recurrence-free survival in the TCGA STLMS cohort, stratified by proteome-based subtypes. Stratification by proteome-based subtypes shows significant survival differences between these subtypes. The P2/P3 subtypes show poorer recurrence-free survival compared to P1.

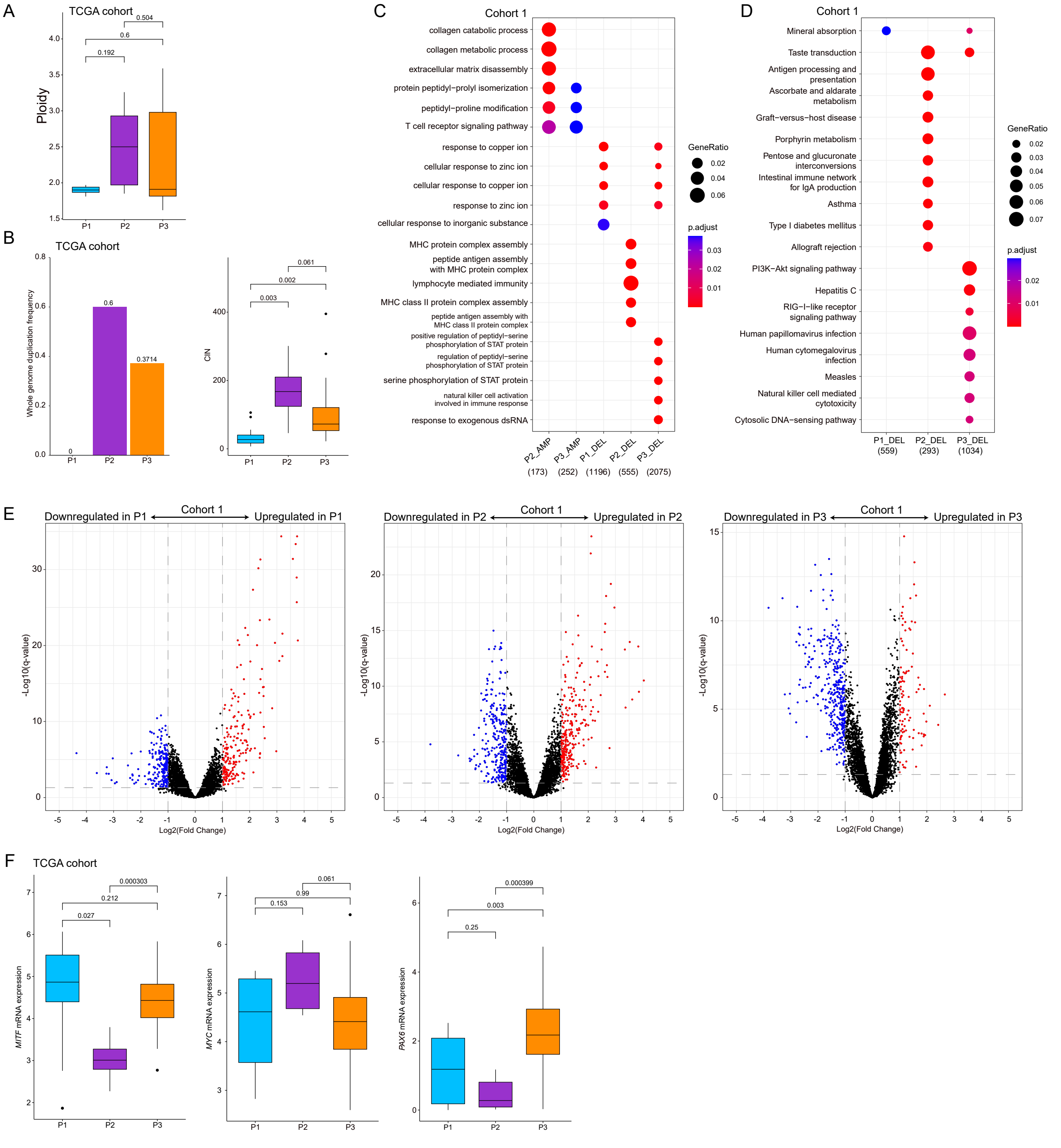

Figure S2. Genomic instability features and regulator profiles in the TCGA STLMS cohort and differential expression analyses of cohort 1, related to Figure 2

(A) Boxplot showing ploidy distributions by inferred proteome subtypes in the TCGA STLMS cohort (n=53).

(B) Bar chart of whole genome duplication frequency by inferred proteome subtypes in the TCGA STLMS cohort (n=53) on the left and boxplot of CIN in the TCGA STLMS cohort (n=52) on the right.

(C) Balloon plot showing GOBP over-representation analysis of genes involved in focal peak amplifications or deletions by subtype in cohort 1.

(D) Balloon plot showing KEGG over-representation analysis of genes involved in focal peak amplifications or deletions by subtype in cohort 1.

(E) Volcano plots showing differentially expressed proteins between subtypes in our cohort 1.

(F) mRNA expression profile of representative regulons by subtypes in the TCGA STLMS dataset.

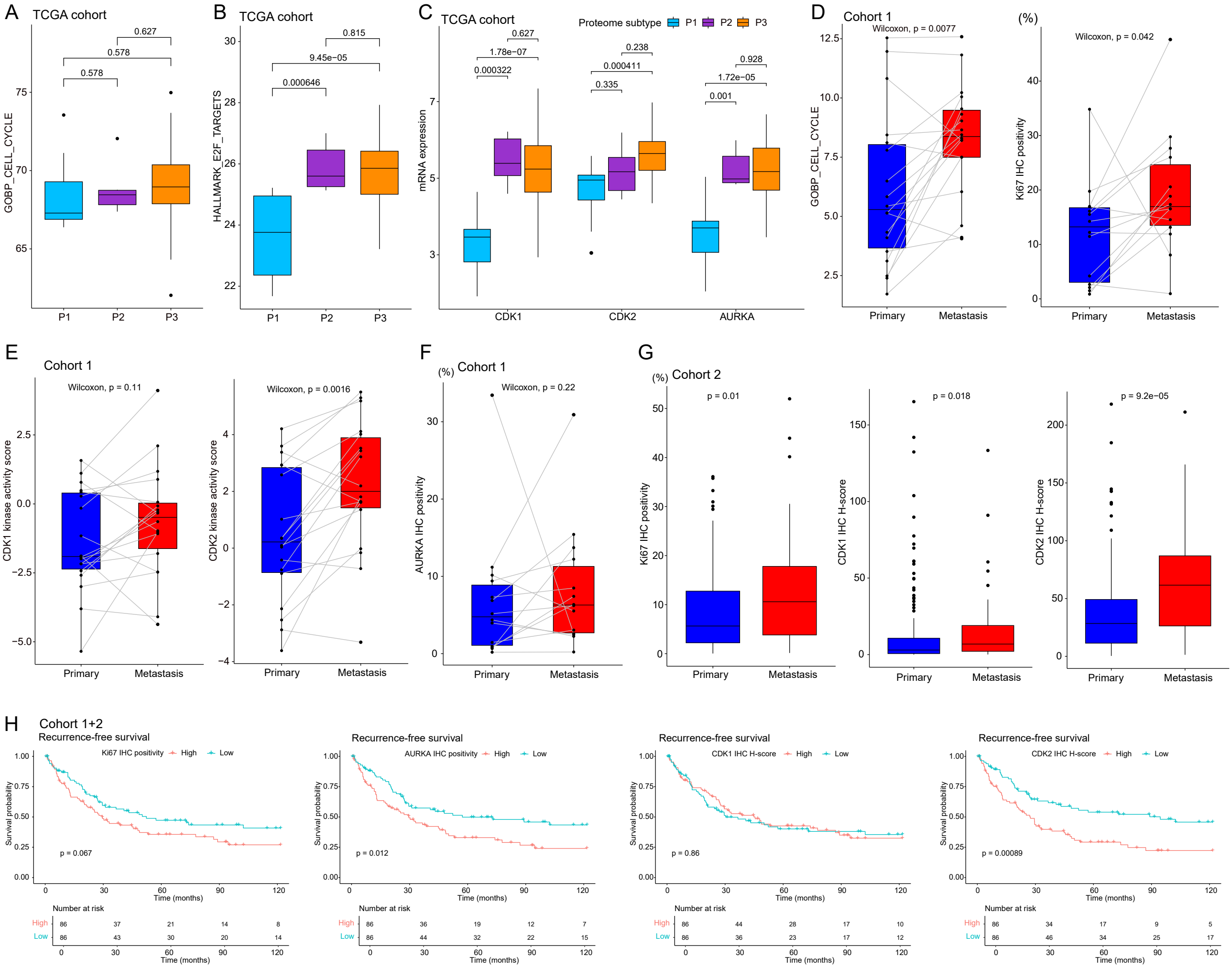

Figure S3. Cell cycle feature differences in the TCGA STLMS transcriptome data and comparison of metastatic samples in cohort 1 against matched primary samples, related to Figure 3

(A) Boxplot showing cell cycle score differences by proteome subtypes in the TCGA STLMS cohort.

(B) Boxplot of "E2F targets" scores of ssGSEA by subtypes in the TCGA STLMS cohort. P2/P3 show significantly higher scores than P1.

(C) Boxplot of mRNA expression of CDK1, CDK2, and AURKA in the TCGA cohort. P2/P3 show significantly higher expressions of CDK1 and AURKA than P1. CDK2 mRNA expression in P3 is higher than in P1.

(D) Boxplot on the left side showing cell cycle score differences between 18 paired primary and metastatic samples in cohort 1. Boxplot on the right side showing Ki67-positive tumor cell frequency based on IHC by subtypes (14 paired samples) in cohort 1.

(E) Boxplot showing kinase activity scores of CDK1/2 between primary and metastatic samples. 18 paired samples were analyzed from cohort 1.

(F) Boxplot of AURKA IHC scores. 14 paired samples were available for assessment.

(G) Boxplot of Ki67, CDK1, and CDK2 IHC scores between primary and metastasis samples in cohort 2, confirming metastatic samples show significant increases of these markers.

(H) Kaplan-Meier analysis of recurrence-free survival in combined cohorts 1 and 2, stratified by Ki67, AURKA median positivities, CDK1/2 IHC H-score median values. Patients with high AURKA positivity or CDK2-high H-scores show poorer outcomes, concordant with poor survival outcome of the P2/P3 subtypes (cell cycle-enriched subtypes).

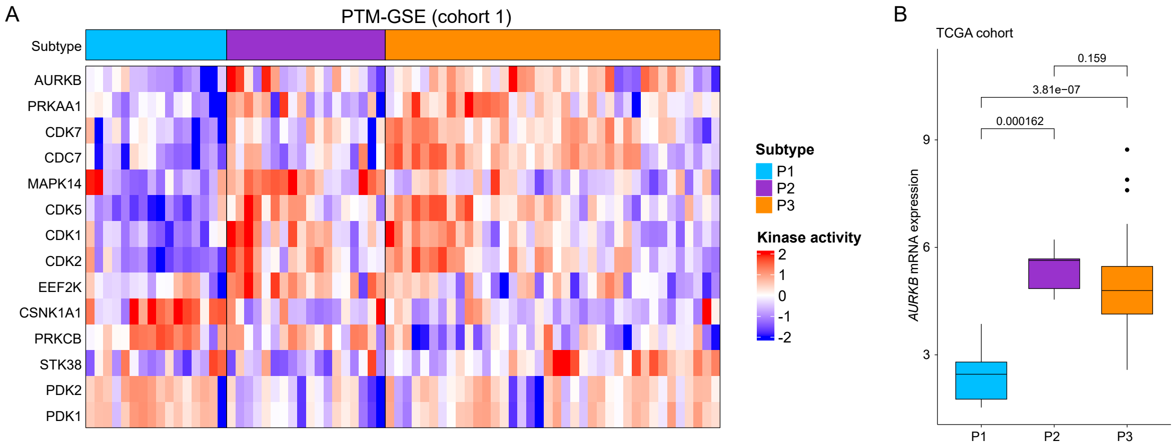

Figure S4. Phosphorylation landscape of cohort 1 and validation of findings in the independent cohort 2 and the external TCGA STLMS cohort, related to Figure 4

(A) A heatmap of PTM-GSE scores. Terms with significant differences between subtypes (ANOVA q<0.01) are shown. AURKB activity is high in P2/P3, consistent with the RoKAI results.

(B) Boxplot of AURKB mRNA expression by subtypes in the TCGA cohort. AURKB mRNA expression is significantly higher in P2/P3 compared to P1.

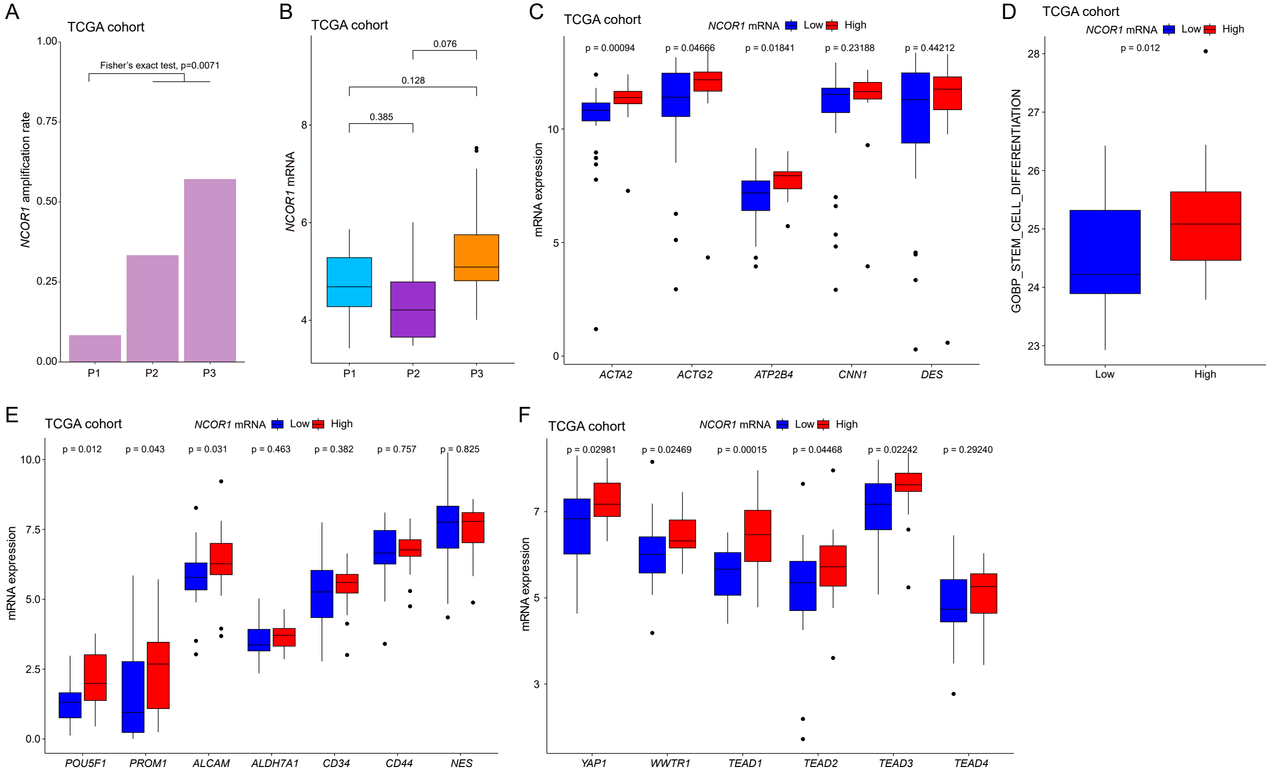

Figure S5. NCOR1 characteristics in the external TCGA STLMS dataset, related to Figure 5
(A) NCOR1 amplification shows higher frequency in P2/P3 compared to P1.
(B) NCOR1 mRNA expression in P3 trends higher than in other subtypes, but not statistically significant.
(C) mRNA expression of smooth muscle markers shows higher levels in the NCOR1-high group.
(D) Stem cell differentiation scores (GOBP ssGSEA scores) are significantly higher in the NCOR1-high group.
(E) mRNA expression of POU5F1, PROM1 (CD133), and ALCAM are significantly higher in the NCOR1-high group.
(F) Boxplots of YAP1/TAZ (encoded by WWTR1) related molecules. mRNA expression of YAP1, WWTR1, and TEAD1/2/3 is significantly higher in the NCOR1-high group.

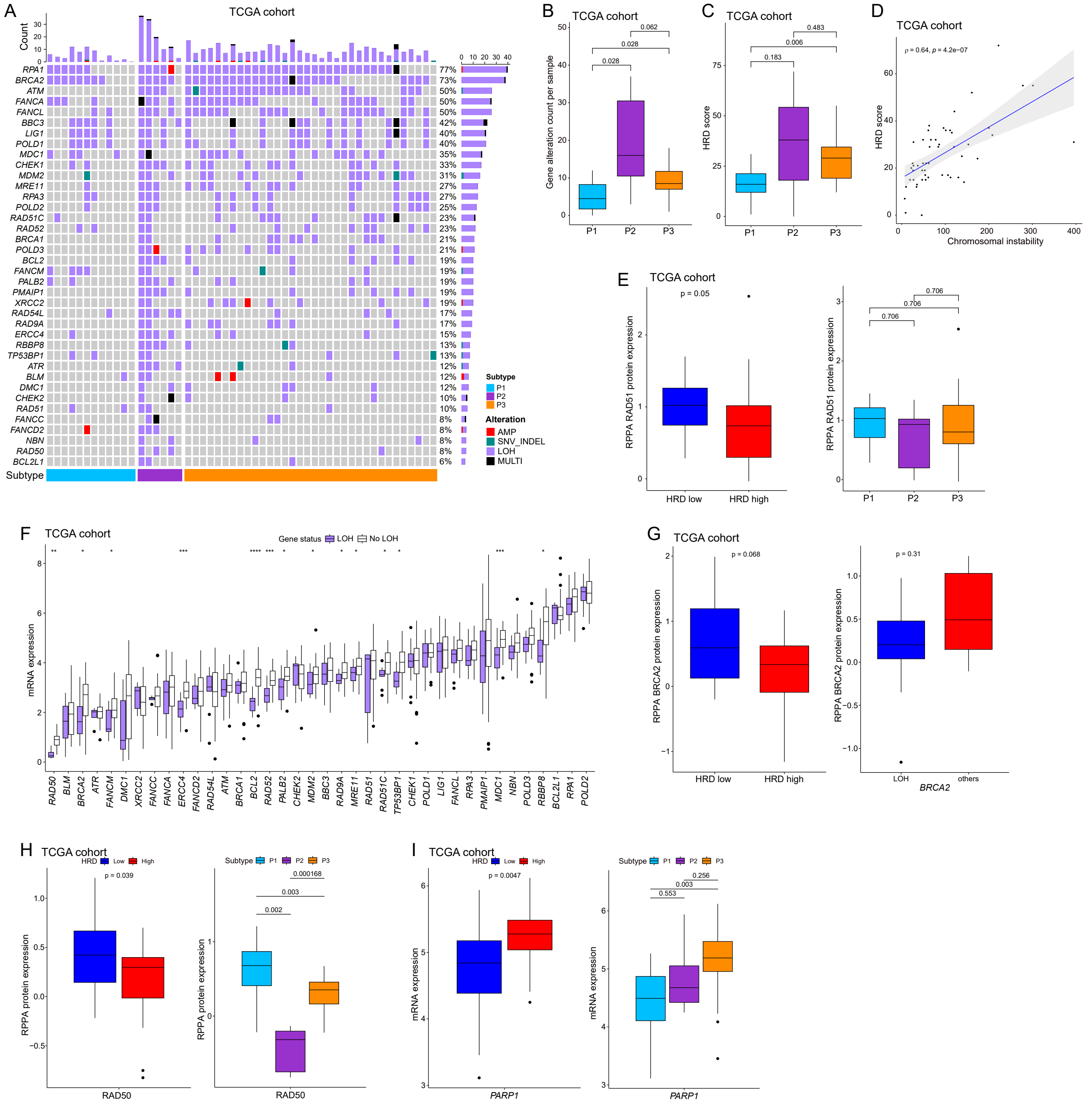

Figure S6. HRR pathway in validation cohort 2 and the external TCGA cohort, related to Figure 6

(A) Oncoprint of HRR pathway coponents showing frequent LOH events in the TCGA STLMS cohort.

(B) Boxplot of genomic alteration counts in the HRR pathway per sample showing significantly higher occurrence in P2/P3 in the TCGA cohort.

(C) The HRD score is higher in P2/P3 compared to P1 in the TCGA cohort.

(D) The HRD score positively correlates with CIN in the TCGA cohort.

(E) The RAD51 protein expression is lower in the HRD-low group and P2/P3 vs. P1, but not significant in the TCGA cohort.

(F) Boxplots of mRNA expression between LOH and non-LOH groups for each HRR gene. All 38 HRR genes are shown. Most genes, including BRCA2, show lower expression in the LOH group compared to the non-LOH group. Note: all genes with statistical significance show expressional decrease in the LOH group in the TCGA cohort.

(G) Boxplots of BRCA2 protein expression between HRD-low and HRD-high groups and BRCA2 LOH and non-LOH groups. BRCA2 protein expression shows the same trend as in cohort 1.

(H) Boxplots of RAD50 protein expression between HRD-low and HRD-high groups and by proteome subtypes. RAD50 protein expression is significantly downregulated in the HRD-low group and in P2/P3 subtypes.

(I) Boxplot of PARP1 mRNA expression between HRD-low and HRD-high groups and by proteome subtypes. PARP1 mRNA expression is significantly higher in the HRD-high group and the P3 subtype.

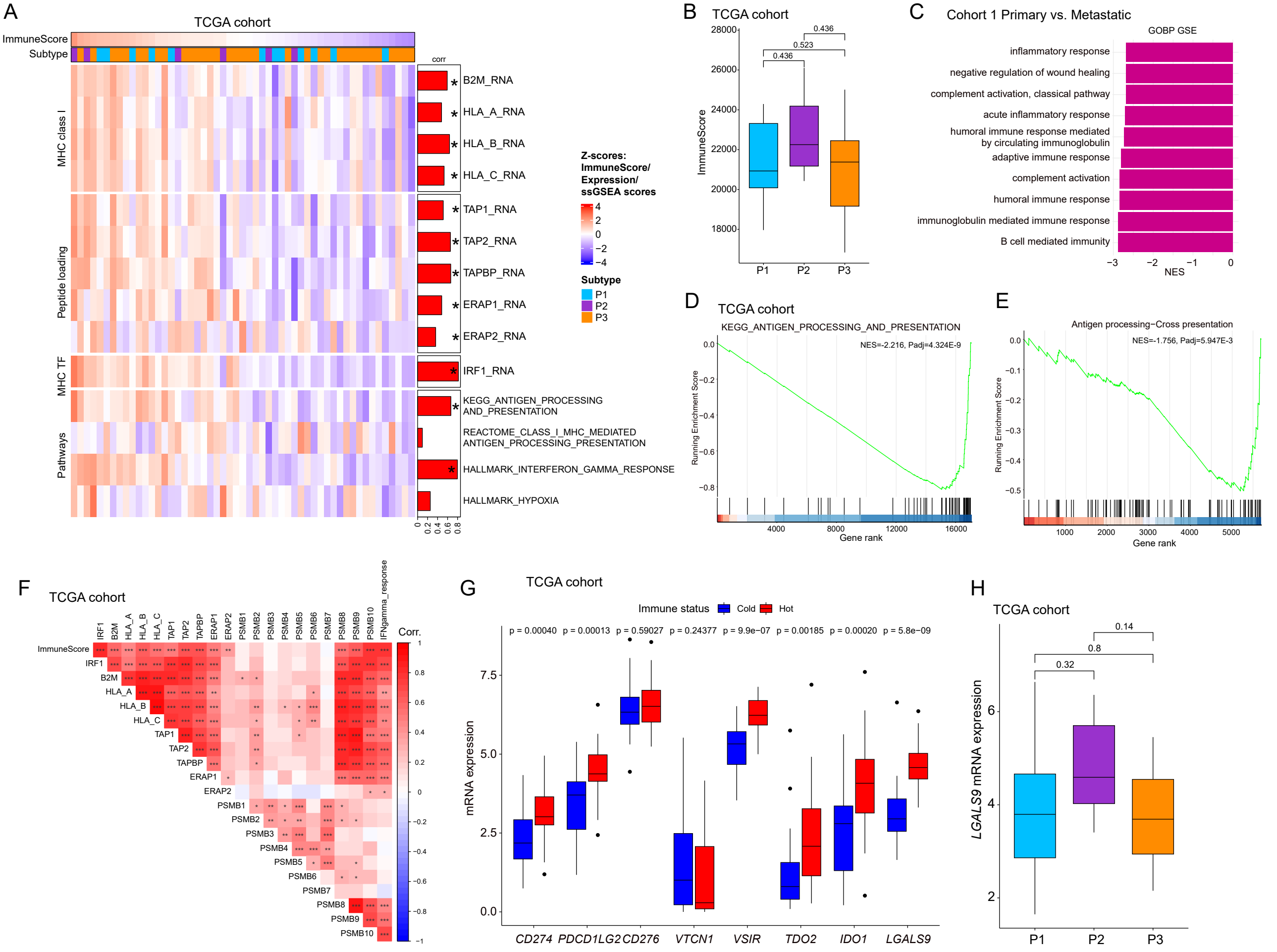

Figure S7. Tumor microenvironment analysis of the external TCGA STLMS dataset and comparison of primary vs. metastatic lesions, related to Figure 7
(A) A heatmap with immune signatures of the TCGA dataset. Immune-cold tumors show low expression of MHC class I molecules and peptide loading molecules, confirming findings in cohort 1. Bars on the right side of the heatmap denote Spearman's correlation coefficients between ImmuneScores and each gene/signature.
(B) Boxplot showing ImmuneScores by inferred proteome subtypes. Although not statistically significant, P3 shows lower scores compared to other subtypes.
(C) Top 10 significantly downregulated GOBP terms. GSEA of differential expression between primary and metastatic tumors in cohort 1 shows significant downregulation of immune-related pathways in metastatic tumors compared to primary tumors.
(D) GSE plot of KEGG pathway analyses showing significant downregulation of antigen processing pathway coponents in immune-cold tumors compared to immune-hot tumors in the TCGA dataset.
(E) GSE plot of Reactome pathway analyses showing significant downregulation of antigen processing pathway components in metastatic tumors compared to primary tumors in cohort 1.
(F) A correlation plot (Spearman's correlation coefficients) including the ImmuneScore, IRF1, and antigen processing machinery protein expression in the TCGA cohort. *, p<0.05; **, p<0.01; ***, p<0.001. Most genes and ImmuneScore show positive correlation with each other. IFNgamma_response scores are ssGSEA scores of the "Hallmark_Interferon_Gamma_Reponse" term.
(G) mRNA expressions of immunosuppressive genes in the TCGA cohort. All genes except CD276 and VTCN1 show significantly higher expression in immune-hot tumors compared to immune-cold tumors.
(H) Boxplot of LGALS9 mRNA expression by subtypes in the TCGA cohort. P2 shows higher expression of LGALS9 compared to P1/P3, which is consistent with the findings in cohort 1.
